# Early Real-World Clinical Outcomes and Astigmatism Vector Analysis of Toric Intraocular Lenses for High Astigmatism (≥2.0 D)

**DOI:** 10.3390/jcm15093343

**Published:** 2026-04-28

**Authors:** Silvia Victoria Prodescu, Paul Filip Curcă, Cătălina Ioana Tătaru, Călin Petru Tătaru

**Affiliations:** 1 Doctoral School, Ophthalmology Department, Carol Davila University of Medicine and Pharmacy, 020021 Bucharest, Romania; silviavpro@gmail.com; 2 Ophthalmology Department, Alcor Clinic, 030836 Bucharest, Romania; catalina_tataru@yahoo.com (C.I.T.); calinpetrutataru@yahoo.com (C.P.T.); 3 Ophthalmology Department, Central Military Emergency University Hospital “Dr. Carol Davila”, Carol Davila University of Medicine and Pharmacy, 010825 Bucharest, Romania; 4 Ophthalmology Department, Clinical Institute for Ophthalmological Emergencies “Prof. Dr. Mircea Olteanu”, Carol Davila University of Medicine and Pharmacy, 010464 Bucharest, Romania; 5 Ophthalmology Department, Clinical Institute for Ophthalmological Emergencies “Prof. Dr. Mircea Olteanu”, 010464 Bucharest, Romania

**Keywords:** toric intraocular lens, high astigmatism, vector analysis, Alpins method, cataract surgery, refractive lens exchange, oblique astigmatism, real-world outcomes

## Abstract

**Background/Objectives**: Toric intraocular lens (IOL) implantation is the standard approach for correcting corneal astigmatism during cataract surgery and refractive lens exchange (RLE). Evidence on outcomes in eyes with high corneal astigmatism (≥2.00 diopters, D), particularly in heterogeneous real-world settings, remains limited. This study evaluated visual, refractive, and astigmatic vector outcomes of toric IOL implantation in a consecutive high-astigmatism cohort and investigated predictors of residual astigmatic error. **Methods**: This single-center, single-surgeon retrospective analysis of prospectively collected data included 161 eyes (118 patients) with preoperative corneal astigmatism ≥ 2.00 D undergoing cataract surgery or RLE with toric IOL implantation (June 2023–December 2025). Primary outcomes at one month included visual acuity, manifest refraction, and Alpins vector analysis at the corneal plane. Secondary analyses comprised refractive stability assessment (*n* = 75 eyes, median seven months), comparison of astigmatic outcomes between emmetropia-targeted and intentional myopia-targeted eyes, and multivariate regression of predictors of residual astigmatic error. **Results**: Mean postoperative UDVA and CDVA were 0.19 ± 0.24 and 0.09 ± 0.15 logMAR, respectively. Spherical equivalent prediction error was −0.19 ± 0.42 D (69.6% within ±0.50 D of target). Mean residual cylinder was 0.52 ± 0.49 D; 62% and 88.8% of eyes achieved ≤0.50 D and ≤1.00 D, respectively. Vector analysis demonstrated a mean difference vector of 0.53 ± 0.44 D, a correction index of 1.04 ± 0.20, and near-zero centroid deviation (0.03 D @ 43°), indicating the absence of systematic directional prediction error. Refractive outcomes were stable at medium-term follow-up. Astigmatic correction accuracy was equivalent between emmetropia-targeted and intentional myopia-targeted eyes (*p* > 0.05 for all primary metrics). Multivariate regression identified IOL cylinder power (β = 0.051, *p* = 0.031) and oblique astigmatism orientation (β = 0.299 vs. WTR, *p* = 0.032) as independent predictors of greater residual astigmatic error. No sight-threatening complications occurred. **Conclusions**: Toric IOL implantation provides safe, predictable, and stable correction of high corneal astigmatism in a real-world mixed cohort. Astigmatic accuracy is maintained regardless of intended spherical refractive strategy, supporting the use of toric IOLs in highly myopic patients targeted for residual myopia. Oblique astigmatism orientation is an independent predictor of reduced correction accuracy, consistent with known limitations of current toric calculators for this meridian.

## 1. Introduction

Refractive errors are among the most frequent treatable causes of visual impairment [1]. Astigmatism is a common refractive error caused by meridional asymmetry in the curvature of the cornea or lens [2], which creates an asymmetric refraction of light rays on a stronger power meridian [2]. Total ocular astigmatism is given by combined corneal astigmatism (unequal curvature of the anterior and/or posterior cornea) and internal astigmatism (unequal curvatures of the front and back surfaces of the crystalline lens, decentration or lens tilt, or unequal refractive indices of the lens) [3]. Corneal astigmatism is differentiated by the orientation in degrees (°) of the strongest power meridian [2] into with-the-rule (60–120°) [4], against-the-rule (0–30° or 150–180°) [4], and oblique astigmatism (in between 30–60° and 120–150°) [4]. Corneal astigmatism is typically regular [5]. Irregular astigmatism occurs when the angle between the axis of maximum curvature and minimum curvature is not at the right angle or when the curvature of a refractive surface is not axially symmetric [5]. Corneal irregular astigmatism often cannot be corrected with spectacles [5]. Several surgical options are available for astigmatism correction.

Laser-assisted corneal refractive surgery [6] is preferred in patients with preserved normal accommodation and a clear crystalline lens. For patients with presbyopia and regular astigmatism, refractive lens exchange (RLE) with toric multifocal or extended depth of focus (EDOF) intraocular lenses (IOLs) offers spectacle independence and simultaneous astigmatism correction [7]. RLE can also be performed in patients with high refractive errors that are at or above the corrective possibilities of laser-assisted refractive surgery, such as high hyperopia [7] or high myopia [8]. Bioptics surgery is the combination of refractive lens exchange and IOL implantation, followed by laser-assisted refractive surgery [9]. Bioptics surgery is reserved for high hyperopia and myopia [9] and can accommodate the use of presbyopia-correcting IOLs to maintain spectacle independence [10].

After uncorrected refractive errors, cataract is the second most common cause of visual impairment [11]. After cataract phacoemulsification or aspiration, the lens can be replaced with a Toric Intraocular Lens (Toric IOL), which effectively corrects regular astigmatism. For irregular astigmatism, toric IOLs can offer partial correction by placement for the higher cylinder power meridian [5], with lesser power meridians either left uncorrected or corrected via additional laser-assisted refractive procedures.

Toric IOL implantation is highly effective and preferred to other astigmatism correction methods in cataract patients. In a systematic review, Yen W. et al. found toric IOL implantation in cataract patients preferred to femtosecond laser-assisted astigmatic keratotomy (FSAK) [12]. Toric IOLs produced less postoperative refractive cylinder and better uncorrected distance visual acuity (UDVA) compared to FSAK [12]. FSAK presented better (smaller) vectorial astigmatism parameters, such as Target-Induced Astigmatism (TIA), Surgically Induced Astigmatism (SIA) and Correction Index (CI) [12].

Toric intraocular lens astigmatism correction is critically dependent upon correct axis placement and the rotational stability of the lens [13]. For every degree that a toric IOL is moved from its intended axis, it loses 3.3% of its astigmatism-correcting effect, with complete loss occurring at 30 degrees off-axis [14,15]. Misalignment is considered the total deviation of the toric IOL from its intended target meridian to its final postoperative axis [13]. Postoperative rotation is the change in axis position from the measured implanted axis to its final postoperative axis [13]. In a single-arm meta-analysis of 51 separate studies, Li et al. verified rotational stability for a variety of toric IOL designs (loop haptic, plate haptic, double-loop haptic, plate double-loop) and reported a mean rotation of 2.36° [13].

Several factors can influence IOL rotational stability, such as high axial myopia [14,16], dimensions of the capsular bag (estimated via white-to-white WTW and lens thickness (LT) parameters) [16], characteristics of the IOL design [16], surgical factors [16] and postoperative capsular aspects [16]. Singh A. et al. reported a correlation between eyes with axial length ≥ 25 mm and an increase in mean rotation of toric IOLs [17]. There is an association between higher axial length and a larger capsular bag [16]. Due to a larger capsular bag size, friction between the capsular bag and the IOL haptic can be reduced, thus making IOL rotation more likely [16]. Several authors recommend prophylactic placement of a capsular tension ring (CTR) in case of high eye axial length [14,17,18,19] or even suturing the IOL to the CTR ring [20]. If toric IOL rotation is significant, then studies recommend repositioning the IOL within 1–3 weeks of the initial surgery [16] with Ma, D. et al. multicenter retrospective study recommending a mean time of 15 days postoperative [21]. Residual astigmatism after toric IOL implantation is most frequently dependent on toric IOL misalignment versus the calculated biometry axis placement [22]. Less frequently, the IOL is oriented as intended; however, preoperative biometry calculations are proven deficient [22]. Higher-strength toric IOLs are associated with an increased margin of error in residual astigmatism [22]. In most cases, repositioning of the toric IOL on the correct axis effectively reduces residual astigmatism [22].

The aim of the present study was to evaluate visual, refractive and astigmatic vector analysis outcomes of toric IOL implantation in a consecutive real-world cohort of patients with high preoperative corneal astigmatism ≥ 2.00 D.

## 2. Materials and Methods

### 2.1. Study Design and Setting

This was a single-center, single-surgeon retrospective analysis of prospectively collected real-world data. All procedures were performed at Clinica ALCOR, a private ophthalmology center in Bucharest, Romania, by the same experienced anterior segment surgeon (Prof. C.P. Tătaru). The study was conducted as part of a doctoral research thesis at Carol Davila University of Medicine and Pharmacy, Bucharest, and adhered to the tenets of the Declaration of Helsinki. Ethics committee approval was obtained from the independent ethics committee of the Clinical Institute for Ophthalmological Emergencies “Prof. Dr. Mircea Olteanu”, Bucharest (1267/20 March 2026), and written informed consent (for both the surgical procedure and study participation) was obtained from all patients. The aim of the study was to analyze the early postoperative assessment (at one month) of visual and refractive outcomes, including vectorial analysis by the Alpins method [23], in a real-world cohort of consecutive eyes with astigmatism magnitude greater than two diopters that underwent toric intraocular lens (IOL) implantation.

### 2.2. Study Inclusion and Exclusion Criteria

**Figure 1:**
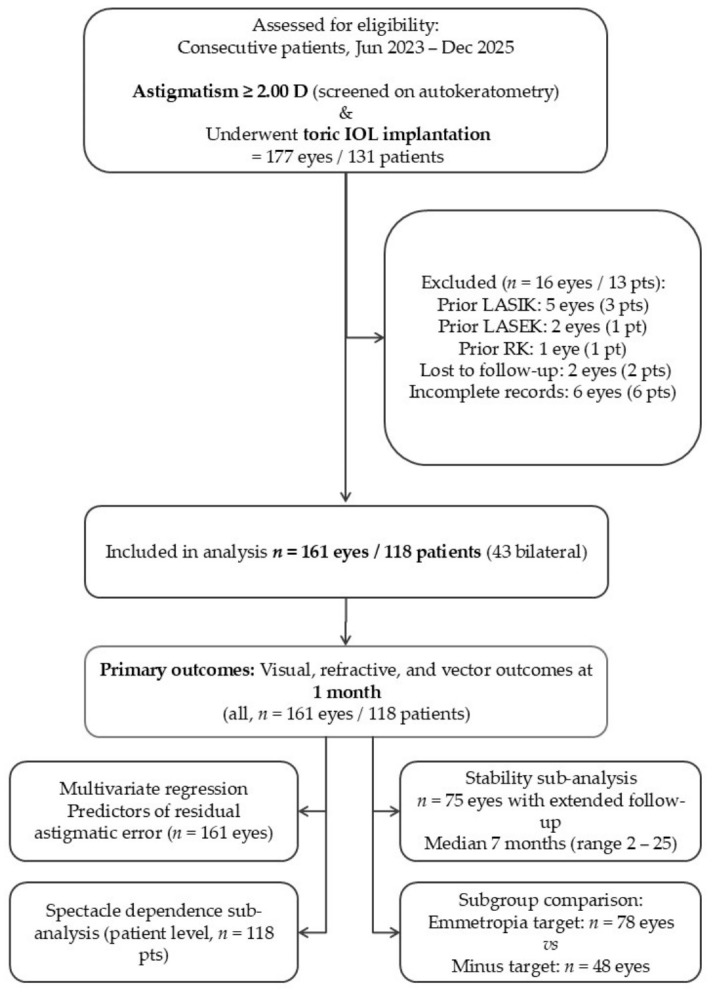
Study Flowchart with patient inclusion and exclusion criteria, analysis synopsis and secondary statistical analysis (multivariate regression, spectacle dependence sub-analysis, stability sub-analysis, subgroup comparisons). Astigmatism ≥ 2.00 D refers to preoperative corneal astigmatism measured by autorefractokeratometry at the time of initial clinical assessment. Inclusion in the final analysis required completion of the 1-month postoperative visit with full clinical and refractive data. Bilateral cases (43 patients, 86 eyes) were included and are acknowledged as a partial study limitation. The stability sub-analysis and subgroup comparison were conducted in predefined subsets of the primary cohort as described in Section 2. D—diopters; IOL—Intraocular Lens; LASIK—Laser-Assisted In Situ Keratomileusis; LASEK—Laser-Assisted Epithelial Keratomileusis; RK—Radial Keratotomy; pt(s)—patient(s); *n*—number of eyes (unless otherwise specified).

Data were prospectively collected from consecutive patients who underwent lens-based surgery, either phacoemulsification cataract extraction or refractive lens exchange (RLE), with toric intraocular lens (IOL) implantation between June 2023 and December 2025. The primary inclusion criterion was a preoperative corneal astigmatism of ≥2.00 D, measured by autorefractokeratometry at the time of initial clinical assessment. The remaining inclusion criteria were age ≥ 18 years and the availability of complete postoperative data at the 1-month visit (Figure 1). Exclusion criteria included refusal to participate, loss to follow-up before the 1-month endpoint (*n* = 2 eyes/2 patients), and incomplete medical records (*n* = 6 eyes/6 patients) (Figure 1). Prior corneal refractive surgery was also an exclusion criterion (*n* = 8 eyes/5 patients). Eyes with ocular comorbidities, including amblyopia, glaucoma, myopic maculopathy, corneal dystrophy, and one case of stable keratoconus grade I in an older patient at low risk of progression, were retained in the analysis, as these conditions did not alter the planned surgical approach or the validity of vector analysis outcomes, and their inclusion reflects the real-world clinical population in whom toric IOL implantation is performed. One case of traumatic cataract with iris irregularity was also included. For the purpose of visual acuity outcome interpretation, eyes were classified post hoc into those with and without preoperatively documented ocular comorbidities expected to limit postoperative visual acuity independently of refractive correction. A sensitivity analysis of postoperative visual acuity restricted to eyes without such comorbidities is reported in Section 3.4.1. In eyes with dense lens opacity precluding adequate pre-operative fundoscopic assessment, concurrent posterior segment pathology could not be reliably excluded preoperatively, and such eyes were therefore classified as non-visual acuity-limiting for the purpose of the sensitivity analysis, as no a priori documentation of visual limitation was available. No randomization was applied; IOL type (plate haptic, hydrophilic; loop haptics hydrophobic), optical design (toric monofocal, toric multifocal, toric extended depth of focus), and refractive target (emmetropia or intentional residual minus) were determined by shared clinical decision-making between patient and surgeon based on individual indication and preference. No monovision strategy was implemented in any case. Because inclusion was based on autorefractokeratometric initial screening, whereas vector analysis used tomo-topographic measurements (Anterion^®^ anterior segment optical-coherence tomograph, Heidelberg Engineering GmbH, Heidelberg, Germany), values may differ slightly, particularly in dense cataract cases where autorefractokeratometry is more challenging to obtain reliably.

### 2.3. Preoperative Assessment

All patients underwent a comprehensive preoperative ophthalmic examination, including measurement of uncorrected distance visual acuity (UDVA) and corrected distance visual acuity (CDVA), manifest refraction, intraocular pressure, slit-lamp biomicroscopy, and fundus examination. Where fundus visualization was not possible due to dense lens opacity, B-scan ultrasonography was performed to exclude posterior segment pathology. When clinically indicated, macular or optic nerve optical coherence tomography (OCT) was also obtained.

Visual acuity was recorded in decimal notation and converted to the logarithm of the minimum angle of resolution (logMAR) and Snellen values for analysis. Preoperative visual acuity values below the measurable Snellen range (counting fingers [CF], hand movements [HM], and light perception [LP]) were assigned for analytical purposes, logMAR equivalents of 1.8, 2.3, and 2.8, respectively, in accordance with commonly used conversion schemes and to ensure consistency with the mEYEstro software version 2.5.0.0 used for graphical outcome analysis. The maximum recorded visual acuity was 1.0 (20/20 Snellen; logMAR 0.0); no values above this threshold were entered into the analysis.

Preoperative manifest refraction data was available in 130 eyes, while in the remaining eyes, dense lens opacity precluded reliable refraction measurement. However, statistical and vector analyses were based on corneal astigmatism measurements obtained via biometry and available in all 161 eyes. This approach allowed the inclusion of advanced cataract cases while maintaining consistency and comparability in outcome analysis.

Ocular biometry was performed using the Anterion^®^ (Heidelberg Engineering, Heidelberg, Germany) swept-source optical coherence tomography biometer, which provided axial length, keratometry, anterior chamber depth, lens thickness, and white-to-white measurements. In cases where optical biometry was not feasible due to dense media opacity, immersion A-scan ultrasonography or an alternative biometer was used: Topcon Aladdin (Topcon Corporation, Tokyo, Japan) or Argos^®^ (Alcon Healthcare, Fort Worth, TX, USA). Keratometric astigmatism for inclusion screening was determined by autorefractokeratometry; however, all vector analyses used tomo-topographic keratometry (K2−K1) from Anterion^®^ as the baseline corneal astigmatism measurement. Anterior chamber depth (ACD) was derived from Aqueous Depth (AQD) measurement from the endothelium plus central corneal thickness (CCT), both parameters measured with Anterion^®^.

Astigmatism was defined as with-the-rule (WTR) when the steep meridian was located in between 60 and 120°, as against-the-rule (ATR) with the steep meridian in between 0–30° or 150–180°, and as oblique in between 30–60° and 120–150°.

### 2.4. IOL Selection and Surgical Technique

**Figure 2:**
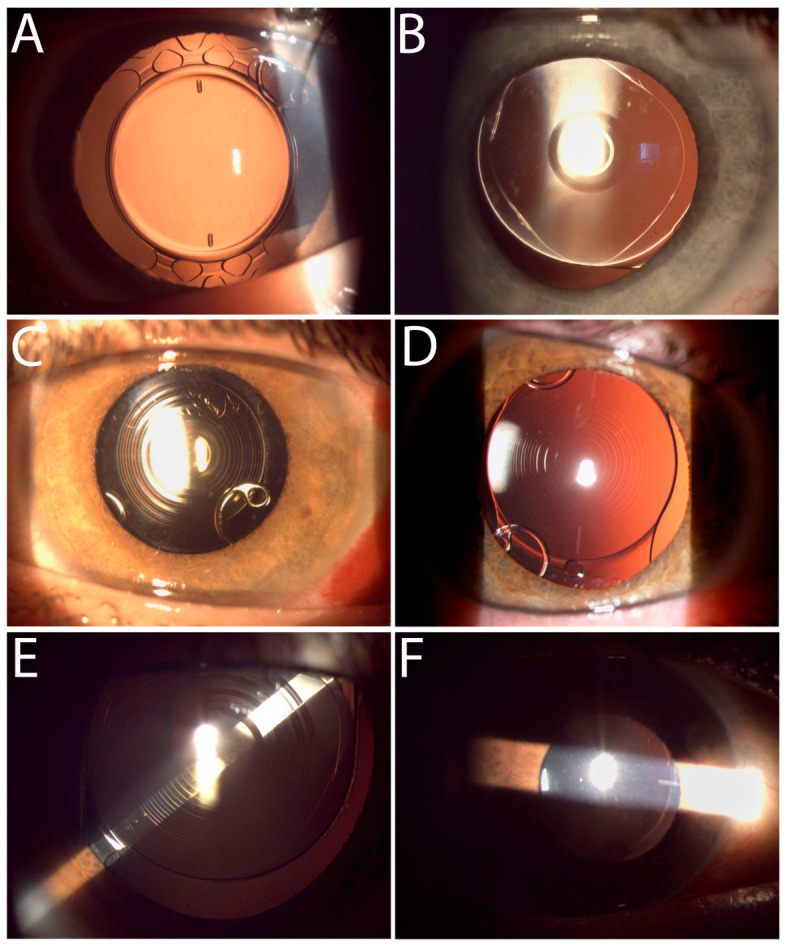
Images of toric IOL types as presented in Section 2, courtesy of Prof. C.P. Tătaru’s archive. (**A**) SIFI Miniwell Toric. (**B**) Alcon AcrySof^®^ IQ Vivity Toric. (**C**) Alcon AcrySof^®^ IQ PanOptix Toric and Cionni Capsular Tension Ring. (**D**) Zeiss AT LISA Tri-Toric 949M/MP and Cionni Capsular Tension Ring. (**E**) AcrySof^®^ IQ PanOptix Toric. (**F**) Zeiss AT TORBI 719M/MP.

Toric IOL cylinder power and alignment axis were calculated using manufacturer-specific or dedicated toric calculators, including the Alcon Toric Calculator (Alcon Laboratories Inc., Fort Worth, TX, USA), the ESCRS Toric IOL Calculator (The European Society of Cataract and Refractive Surgeons, London, UK), and the Zeiss Toric Calculator (Carl Zeiss Meditec AG, Jena, Germany). IOL power was determined using the Kane formula (44.1% of eyes), the Barrett Universal II formula (31.1%), or the Zeiss proprietary formula (24.7%). The selection of the calculator and formula was at the surgeon’s discretion based on biometric data quality and IOL type. The toric IOL the eyes in the study cohort received and respective proportions are as follows (Figure 2): monofocal Alcon AcrySof^®^ IQ Toric SN6ATx, 54.7% (Alcon Laboratories Inc., Fort Worth, TX, USA); Alcon Clareon^®^ Toric IOL CNW0Tx, 11.8% (Alcon Laboratories Inc., Fort Worth, TX, USA); Zeiss AT TORBI 709M/MP or 719M/MP, 12.4% (Carl Zeiss Meditec AG, Jena, Germany) and presbyopia-correcting Zeiss AT LISA tri toric 949M/MP, 12.4% (Carl Zeiss Meditec AG, Jena, Germany); Alcon AcrySof^®^ IQ Vivity Toric DFTx15, 4.3% (Alcon Laboratories Inc., Fort Worth, TX, USA); Alcon AcrySof^®^ IQ PanOptix Toric TFNTx0, 2.5% (Alcon Laboratories Inc., Fort Worth, TX, USA); SIFI Miniwell Toric, 1.9% (SIFI Medtech, Catania, Italy).

All procedures were performed under topical (the majority of cases) or retrobulbar anesthesia (selectively used in dense cataract, expected prolonged surgical time, or reduced patient cooperation cases) by the same surgeon in the same operating theater using the same phacoemulsification platform, Centurion^®^ Vision System (Alcon Healthcare, Fort Worth, TX, USA). Toric axis marking was performed preoperatively at the slit lamp using a surgical marker. The standard surgical technique comprised a 2.2 mm clear corneal main triplanar incision (placed at 170–180° in most cases (0–10° in left eyes), or on-axis in selected cases with favorable astigmatism orientation). A secondary paracentesis was performed, and a continuous irrigation maintainer was placed at the inferior limbus. Continuous curvilinear capsulorhexis, hydrodissection and hydrodelineation, phacoemulsification, irrigation and aspiration of cortical material, and implantation of the toric IOL via a cartridge-based injector aligned to the calculated axis were performed. Both dispersive and cohesive viscoelastics were used; all viscoelastic material was aspirated at the end of the procedure. Wounds were hydrosutured, and the patient was discharged with topical antibiotic, steroid and artificial tears eye-drops treatment plan and a sterile bandage until the following day.

In selected cases, additional intraoperative procedures were performed as clinically indicated: pupilloplasty in one case of traumatic cataract with iris irregularity, intravitreal anti-VEGF injection in two cases with pre-existing macular pathology, and synechiolysis between the iris and anterior lens capsule in one eye with uveitic sequelae. One case of posterior capsule rupture was managed with anterior vitrectomy; the planned toric IOL was successfully implanted in the capsular bag in this case. In one eye, a corneal suture was placed at the main incision at the surgeon’s judgment to ensure wound stability; this was not considered a complication.

In eyes with zonular weakness or high axial length, a capsular tension ring (CTR) was implanted prior to IOL insertion to optimize capsular bag stability and IOL centration (*n* = 28 eyes, 17.4%).

### 2.5. Postoperative Follow-Up and Primary Endpoint

Patients were examined on the first postoperative day, at 3–4 days, and at 1 month (Figure 2). The 1-month visit constituted the primary data collection endpoint and included measurement of UDVA, CDVA, manifest refraction, assessment of spectacle dependence (defined based on records of issuance of optical correction prescription), and evaluation for complications. Patients with postoperative corneal edema or intraocular pressure elevation were reviewed more frequently as clinically required. A subgroup of patients had extended follow-up data available beyond 1 month (*n* = 75 eyes, median 7 months, range 2–25 months); these data were used for a refractive stability sub-analysis.

### 2.6. Outcome Measures

Visual and refractive outcomes were assessed at the primary endpoint of 1 month postoperative and included UDVA, CDVA, manifest spherical equivalent (SE), and residual refractive cylinder. Spherical Equivalent is calculated as Sphere + ½ Cylinder, in diopters. Refractive stability was assessed in the extended follow-up subgroup as the change in SE between the 1-month visit and the most recent available follow-up.

Safety index was defined as the ratio of postoperative CDVA to preoperative CDVA, calculated using decimal visual acuity values. Given that preoperative CDVA in this cohort was frequently reduced by lens opacity rather than refractive error alone, safety index values may be overestimated and should be interpreted with caution, as they primarily reflect visual rehabilitation rather than purely refractive safety.

Efficacy index is defined as the ratio of postoperative UDVA to preoperative CDVA, calculated using decimal visual acuity. However, in cataractous populations, the classic efficacy index is substituted by the comparison of postoperative UDVA to postoperative CDVA, as per current reporting standards and software calculation for cataract surgery refractive outcomes. This approach accounts for the limited relevance of preoperative visual acuity in cataract populations and reflects the relationship between achieved uncorrected and best-corrected postoperative vision. Furthermore, because a proportion of eyes was intentionally targeted for residual myopia, the efficacy index interpretation should also be interpreted guardedly.

Spectacle dependence was assessed at 1 month at the patient level. Patients were classified as fully spectacle independent, or as requiring spectacles for distance only, near only, or both distance and near, based on the spectacle prescription documented in the medical record at the 1-month visit. Achievement of the intended functional outcome was also assessed as a strategy-specific success metric: for minus-targeted monofocal eyes, success was defined as distance-only spectacle use (preserving uncorrected near and intermediate vision); for emmetropia-targeted monofocal eyes, success was defined as near-only spectacle use; and for presbyopia-correcting IOL eyes, success was defined as complete spectacle independence.

### 2.7. Vector Analysis Methodology

Astigmatic vector analysis was performed according to the Alpins method [23] on the early postoperative 1-month data, implemented in Microsoft Excel (Microsoft Corporation, Redmond, WA, USA). Preoperative corneal astigmatism (K2−K1 from Anterion^®^ keratometry) was used as the baseline measurement. Target and postoperative manifest refractive astigmatism values, originally recorded at the spectacle plane (vertex distance (VD) 12 mm), were converted to the corneal plane (vertex distance 0 mm) prior to analysis using the standard vertex distance formula applied to each principal meridian: Dc = Ds/(1 − d × Ds), where Dc is the power at the corneal plane, Ds is the power at the spectacle plane, and d is the vertex distance in meters (0.012 m). The cylinder axis was not altered. This transformation is the recommended methodology for toric IOL outcome analysis and ensures that preoperative corneal astigmatism, target astigmatism, and postoperative refractive astigmatism (and the derived vectors) are all expressed at the same reference plane for vector analysis, consistent with the refractive outcome analysis tools’ (described below) approach, allowing direct comparison between keratometric and refractive astigmatism.

The following vector metrics were derived: target-induced astigmatism (TIA), representing the astigmatic correction the surgery was designed to achieve; surgically induced astigmatism (SIA), representing the actual astigmatic change produced; difference vector (DV), representing the vectorial residual between the TIA and SIA and serving as the primary measure of astigmatic prediction error; correction index (CI), defined as the ratio of SIA to TIA (ideal value = 1.0); index of success (IOS), defined as the ratio of DV to TIA (ideal value = 0); and angle of error (AE), defined as the angular difference between the axis of the SIA and the axis of the TIA (positive values indicating counterclockwise, negative values clockwise deviation).

Graphical representation of astigmatic vectors and prediction error was performed using double-angle plots, generated using The American Society of Cataract and Refractive Surgery’s Astigmatism Double Angle Plot Tool (ASCRS Plot Tool) v1.32 [24]. Double-angle plots display astigmatic data after doubling the axis values to transform astigmatism into true Euclidean vectors, enabling accurate statistical characterization of the centroid and spread of the data as recommended by current standards [24,25,26]. Boxplot representations of refractive astigmatism prediction error and spherical equivalent prediction error were generated using Eyetemis Analysis Tool introduced in the paper published by Kan-Tor et al. [27] according to Reitblat, O. et al. [28] (available at https://www.eyetemis.com (accessed on 24 March 2026), endorsed by ESCRS). Prediction error was defined as the vector difference between the actual postoperative refractive astigmatism and the predicted postoperative refractive astigmatism derived from the toric IOL calculator for each individual eye. Standard refractive outcome graphs (efficacy, safety, spherical equivalent accuracy, cylinder distribution, and stability) were generated using mEYEstro software as presented by Gauvin M. and Wallerstein A. [29].

**Figure A1:**
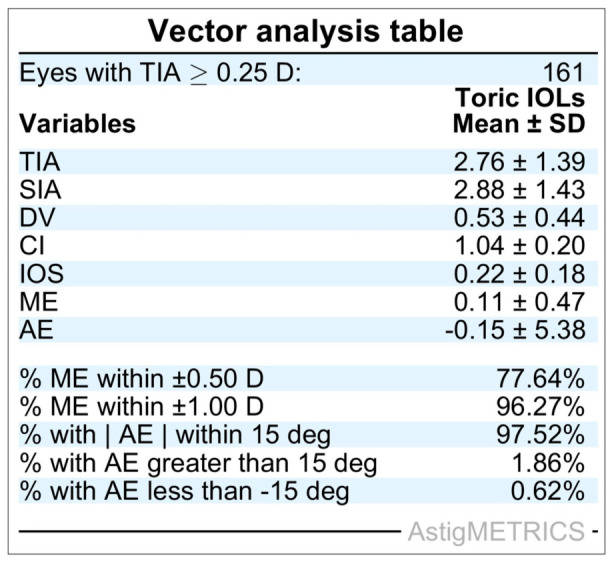
Astigmatic vector analysis output generated by AstigMETRICS software version 1.0.0.0 [30] for the full study cohort (*n* = 161 eyes with TIA ≥ 0.25 D). Results are presented as mean ± standard deviation. Values are consistent with those obtained by custom Excel-based Alpins calculations [23] reported in the main text (Table 5), confirming the internal validity of the vector analysis. Abbreviations: TIA, target-induced astigmatism; SIA, surgically induced astigmatism; DV, difference vector (D); CI, correction index (SIA/TIA); IOS, index of success (DV/TIA); ME, magnitude of error (SIA magnitude—TIA magnitude); AE, angle of error (degrees); SD, standard deviation; D, diopters; deg, degrees.

Vector calculations were independently verified using AstigMETRICS software (version 1.0.0.0) (as presented by Gauvin M. and Wallerstein A. [30], which yielded consistent results; the AstigMETRICS output is provided in the Figure A1.

A sensitivity analysis of primary vector analysis outcomes was performed, excluding eyes with intraoperative events (posterior capsule rupture and corneal suture; *n* = 159), to assess the robustness of the findings. The results are presented in Table A2.

### 2.8. Statistical Analysis

Descriptive statistics for continuous variables are presented as mean ± standard deviation (SD), and median with interquartile range (IQR); categorical variables are reported as frequencies and percentages. All statistical analyses were performed using Minitab version 20.3 64-bit (Minitab LLC, State College, PA, USA) and Microsoft Excel (Microsoft Corporation, Redmond, WA, USA). Missing data was not imputed; analyses were performed on available data.

For the multivariate regression analysis of predictors of residual astigmatic error, candidate variables were first screened by univariate linear regression; those achieving *p* < 0.20 were entered into the multivariate model. Variables screened included preoperative corneal astigmatism magnitude, axial length, age, IOL cylinder power, capsular tension ring use, surgery type, astigmatism orientation, and intended refractive target (Table A1). Regression assumptions were evaluated by inspection of residual plots. A mild right skew of residuals was observed, consistent with the bounded non-negative nature of the DV magnitude outcome variable; given the sample size of 161 eyes, linear regression is generally robust to such minor deviations. Visual inspection did not reveal major deviations from linearity or evidence of misspecification. The Spearman rank correlation was additionally calculated as a non-parametric sensitivity analysis for the key predictor identified in the multivariate model.

For the between-group sub-analysis comparing emmetropia-targeted and minus-targeted monofocal IOL eyes, continuous variables were compared using the Mann–Whitney U test, given the expected non-normal distribution of astigmatic outcome metrics. The angle of error was compared using the independent samples *t*-test, as this signed variable is approximately normally distributed. Categorical variables were compared using the chi-square test or Fisher’s exact test as appropriate. No adjustment for multiple comparisons was applied to this sub-analysis, given its exploratory nature.

To address the clinical distinction between cataract surgery and refractive lens exchange (RLE), a subgroup analysis comparing primary visual, refractive, and astigmatic vector outcomes between the two surgical indication groups was performed. Continuous variables were compared using the Mann–Whitney U test, angle of error was compared using the independent samples *t*-test, and categorical variables were compared using Fisher’s exact test. No adjustment for multiple comparisons was applied, given the exploratory nature of this subgroup analysis.

### 2.9. Limitations

The primary limitations of this study reflect its real-world, non-randomized design. First, extended follow-up data beyond the 1-month primary endpoint were not available for all eyes; a stability sub-analysis was therefore conducted in the subgroup with longer follow-up (*n* = 75 eyes).

Second, a heterogeneous range of toric IOL models and optical designs was used, reflecting genuine clinical practice but limiting direct comparisons between devices.

Third, preoperative manifest refraction was unavailable in a subset of eyes with dense cataract, necessitating the use of corneal astigmatism alone as the baseline vector metric and rendering standard safety and efficacy index calculations fallible from a strictly refractive point of view in eyes with severely reduced preoperative CDVA due to lens opacity.

Fourth, CDVA and UDVA in eyes with concurrent amblyopia, macular pathology, or glaucoma may underrepresent the true refractive accuracy achieved. A separate analysis of visual outcomes was conducted for the subgroup of eyes without pre-operatively documented conditions expected to limit postoperative visual acuity, independent of refractive outcome.

Fifth, bilateral cases (43 patients, 36.4%) represent a partial contravention of the statistical independence assumption; this is acknowledged as a limitation of the regression analysis.

Sixth, systematic IOL rotation data were not available for all cases; toric misalignment was documented in only two eyes where rotation ≥ 15° was clinically identified.

Finally, the inclusion of both emmetropia-targeted and residual myopia-targeted eyes within the same cohort introduces heterogeneity in refractive endpoints; a dedicated sub-analysis comparing these groups was therefore performed.

## 3. Results

### 3.1. Patient Demographics and Baseline Characteristics

Table 1 presents demographics and baseline characteristics of the cases. The mean age at surgery was 64.1 ± 15.1 years (with a median of 69.1 years, range 23–95), and 69 patients (58.5%) were female. The age distribution was left-skewed, reflecting the inclusion of younger patients undergoing refractive lens exchange (RLE). Most patients underwent unilateral surgery (63.6%), while 36.4% contributed to both eyes.

The majority of eyes underwent cataract surgery (82.6%), with the remaining 17.4% undergoing refractive lens exchange (RLE). An emmetropic target was planned in 70.2% of eyes, whereas 29.8% were intentionally targeted for residual myopia (high myopic patients).

### 3.2. Preoperative Data

Preoperative corneal astigmatism had a mean magnitude of 2.66 ± 1.23 D, with a median of 2.37 D (interquartile range [IQR], 1.93–3.08 D). High astigmatism (≥3.0 D) was present in 26.1% of eyes. The distribution of corneal astigmatism was moderately right-skewed. Regarding astigmatism orientation, with-the-rule (WTR) astigmatism was most common (52.2%), followed by against-the-rule (ATR) (40.4%) and oblique astigmatism (7.5%) (Table 1).

Preoperative biometric characteristics are summarized in Table 2. Mean keratometry values were 42.67 ± 1.83 D and 45.33 ± 1.97 D for K1 and K2, respectively, corresponding to a mean corneal astigmatism of 2.66 ± 1.23 D. The mean axial length was 24.73 ± 2.40 mm, with 23.6% of eyes having an axial length greater than 26 mm. Anterior chamber depth averaged 3.47 ± 0.58 mm. Central corneal thickness, lens thickness, and white-to-white measurements were available in most eyes, with a small proportion of missing values due to measurement limitations in dense cataracts.

### 3.3. Surgical Data

Table 3 summarizes surgery-related descriptors. Most eyes received monofocal toric IOLs (78.9%), while 21.1% received presbyopia-correcting toric IOLs (trifocal or EDOF). The most frequently implanted model was AcrySof IQ Toric (54.7%), followed by Clareon Toric and AT TORBI. Toric IOL cylinder power was most commonly in the >2.0 to 3.0 D range (60.2%), with 33.5% of eyes receiving > 3.0 D cylinders. A capsular tension ring was used in 17.4% of eyes. The main corneal incision was typically temporal or near-horizontal (82.0%), with 18.0% of eyes receiving on-axis incisions. Additional intraoperative procedures were infrequent and included intravitreal anti-VEGF injection (1.2%), pupilloplasty (0.6%), and corneal suturing at the main incision (0.6%). IOL cylindrical power calculation was performed using Kane (47.2%), Barrett (28%), or the Zeiss calculator (24.8%) toric formulas.

### 3.4. Visual and Refractive Outcomes

**Figure 3:**
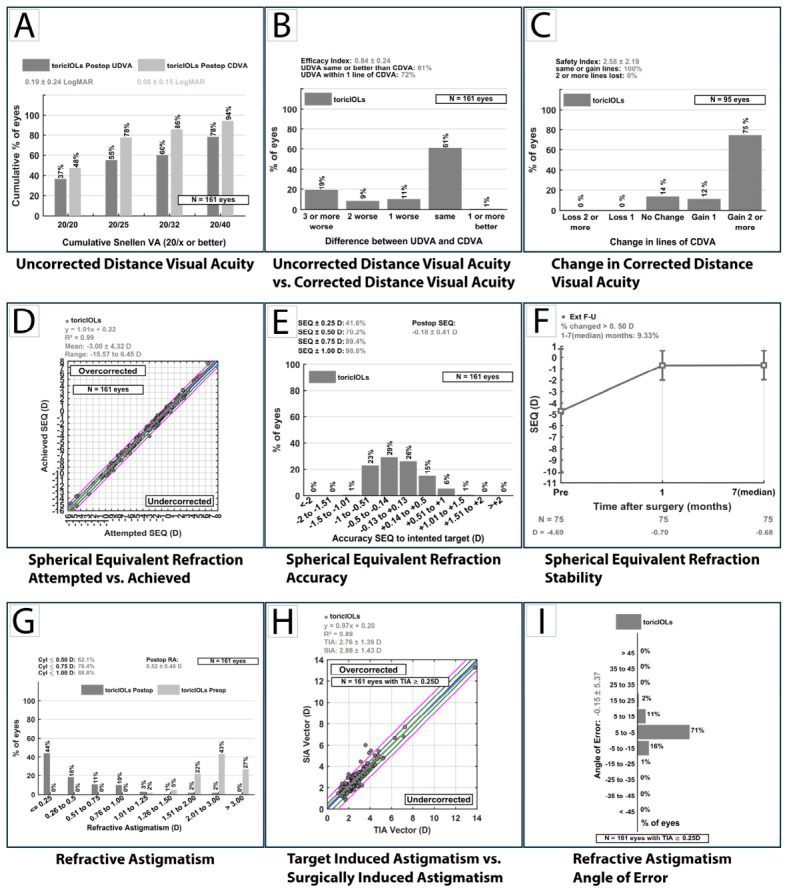
Nine standard graphs for reporting refractive and visual outcomes. Graphs (**A**–**E**) and (**G**–**I**) present primary analysis of the one month postoperative outcomes for the entire study cohort (*n* = 161 eyes); Graph (**F**)—Spherical Equivalent Refraction Stability presents data between two analysis points: primary analysis at one month and extended follow-up (median seven months) for the subset of eyes with available data at both analysis points (*n* = 75 eyes). (**A**) Cumulative Snellen Uncorrected distance visual acuity (UDVA). (**B**) UDVA vs. corrected distance visual acuity (CDVA). (**C**) Changes in CDVA (surgical safety). (**D**) Attempted vs. achieved spherical equivalent refraction (SE). (**E**) SE Refraction accuracy. (**F**) SE Refraction stability over time. (**G**) Refractive astigmatism (**H**) Target induced astigmatism (TIA) vs. Surgically induced astigmatism (SIA). (**I**) Refractive astigmatism angle of error. Generated with mEYEstro version 2.5.0.0 [29]. UDVA, Uncorrected distance visual acuity; CDVA, Corrected distance visual acuity; SE or SEQ, Spherical equivalent; TIA, Target induced astigmatism; SIA, Surgically induced astigmatism; D, Diopter; IOL, Intraocular lens, *n*, number of; logMAR, Logarithm of the minimum angle of resolution; RA, Refractive astigmatism; Ext F-U, Extended follow-up.

As a methodological consideration, CF/HM/LP visual acuities (66 eyes) were converted to logMAR equivalent (1.8, 2.3, and 2.8, respectively) as stated in the Methods section above. These low pre-operative visual eyes, reflecting the presence of advanced cataract eyes in our cohort, were excluded from the computation of the changes in lines of CDVA histogram (Figure 3C) with the mEYEstro software [29], given the fact that Snellen line conversion could not be reliably applied. Furthermore, Table 4 presents refraction data at the spectacle plane (spherical and cylindrical power, spherical equivalent, at VD = 12 mm) for the total of eyes with assessable pre-operative refraction (*n* = 130). However, for the standard graphical representations in Figure 3 (as well as for the vector analysis), corneal astigmatism (k-readings measured with ANTERION^®^) was used (*n* = 161 eyes, topography available for the whole cohort) at the corneal plane (VD = 0 mm). Minor differences between values presented in tables and those shown in standard graphs may arise from rounding conventions applied by the graph-generating software.

#### 3.4.1. Visual Outcomes

Uncorrected distance visual acuity improved from 1.29 ± 0.67 logMAR preoperatively to 0.19 ± 0.24 postoperatively, while corrected distance visual acuity improved from 0.55 ± 0.51 to 0.09 ± 0.15 logMAR. At the Snellen lines level, 37% of eyes achieved UDVA of 20/20 and 48% achieved CDVA of 20/20; at 20/25, these proportions were 55% and 78%, respectively (Figure 3A). The observed gap between UDVA and CDVA distributions is in part attributable to the 29.8% of eyes intentionally targeted for residual myopia, where uncorrected distance visual acuity was not the primary refractive endpoint. Furthermore, post-operative CDVA values also reflect the inclusion of eyes with amblyopia or preexisting ocular pathology (as expected in a real-world setting). Consequently, such outcomes are not solely dependent on achieved refractive error surgical correction, and a separate analysis of visual acuities in eyes without documented visually limiting comorbidities was conducted. Of 161 eyes, 35 (21.7%) had one or more preoperatively documented ocular comorbidities with potential to limit postoperative visual acuity independently of refractive correction, including amblyopia, nystagmus, pathologic myopia with macular changes, posterior segment pathology with known visual-limiting potential, glaucomatous optic neuropathy, uveitic sequelae and visually significant corneal dystrophy or opacity; some eyes had overlapping conditions. In eyes with dense lens opacity precluding adequate preoperative fundus assessment, concurrent posterior segment pathology could not be reliably excluded preoperatively, and these eyes were not classified as visual acuity-limiting for the purpose of this sensitivity analysis because no a priori documentation of reduced visual potential was available. In the 126 eyes without known pre-existing ocular pathology likely to limit postoperative visual acuity (Table 4), mean postoperative UDVA was 0.11 ± 0.15 logMAR (median 0.05) and mean postoperative CDVA was 0.03 ± 0.05 logMAR (median 0.00). Intentional myopia-targeted eyes were present in both subgroups, and postoperative UDVA in minus-targeted eyes reflects the intended refractive strategy. Postoperative visual acuity in eyes with visually significant comorbidities reflects the combined effect of refractive correction and pre-existing ocular pathology, whereas vector analysis metrics, being independent of visual acuity, were not affected by their inclusion in the primary analysis. A cautious interpretation of primary postoperative UDVA vs. CDVA is thus advised in order not to underestimate the functional visual performance in such cases.

#### 3.4.2. Efficacy and Safety Indexes

In this cataract-dominant cohort, the classical definitions of safety and efficacy indices have limited interpretability, as preoperative visual acuity is frequently reduced by lens opacity rather than refractive error. Therefore, the reported indices primarily reflect visual rehabilitation rather than refractive accuracy. However, as per standard refractive outcomes reporting, safety and efficacy indexes are reported in Table 4 and Figure 3B,C, but should not be interpreted as primary indicators for the whole cohort, given the heterogeneity of the real-world study population. Thus, the safety index (SI) was calculated for 95 eyes with measurable preoperative Snellen acuity, excluding the 66 eyes with preoperative CDVA documented as counting fingers, hand motion, or light perception, where line-based comparisons are not applicable. No eye experienced a clinically significant loss of CDVA (≥2 lines). A total of 75% of eyes gained two or more lines of CDVA and 12% gained one line, yielding a mean safety index of 2.58 ± 2.19 (Figure 3C). Such inflated SI is explainable by the substantial visual rehabilitation achieved in eyes with advanced cataract-related visual impairment (in the context of a cohort which includes 82.6% cataractous eyes vs. 17.4% RLE cases; Table 1). The efficacy index represented in Figure 3B (assessed in accordance with outcome reporting convention for cataractous population, comparing postoperative UDVA and postoperative CDVA, *n* = 161) is 0.84 ± 0.24. However, it is important to interpret postoperative UDVA distributions in the context of intentional myopic targeting in some of the cases (29.8%; Table 1), which yield lower uncorrected distance visual acuity despite satisfactory refractive outcomes (see also Section 3.9, where a detailed comparison of visual and refractive outcomes between emmetropia-targeted and myopic-targeted eyes is presented). As such, in addition to the pooled EI, we separately calculated EI in emmetropia-target eyes only (Table 4). Accordingly, the EI was calculated in the 113 eyes with an emmetropic refractive target, and in this subgroup, 87% of eyes achieved postoperative UDVA equal to or better than their postoperative CDVA (EI for cataract surgery), corresponding to a mean efficacy index of 0.87 ± 0.21 (median 1).

The values of both indices reflect the substantial visual rehabilitation achieved in a cohort with predominantly poor preoperative acuity due to advanced cataract, but do not necessarily validate its refractive outcomes strictly. The following refraction-based results are of more clinical value in interpreting the performance of refractive correction than metrics derived from visual acuities in cataract cohorts.

#### 3.4.3. Spherical Equivalent

The mean measured postoperative spherical equivalent (SE) (shown in Table 4) was −0.68 ± 1.29 D, reflecting the inclusion of eyes with intentional myopic targets. Refractive accuracy relative to the intended target (Table 4 and Figure 3E) was high, with 69.6% of eyes within ±0.50 D and 98.1% within ±1.00 D. The SE histogram (Figure 3E) is centered slightly myopic, with very few outliers and the attempted versus achieved SE scatterplot demonstrates a regression slope of 1.01 (R^2^ = 0.99; Figure 3D), confirming excellent spherical predictability across the full range of attempted corrections (−15.57 to +6.45 D). The mean prediction error (relative to expected outcome) was −0.19 ± 0.42 D, indicating a slight overall myopic shift but with good refractive predictability. The mild myopic shift might be consistent with the substantial proportion of long eyes in this cohort (axial length > 26 mm in 23.6% of eyes; Table 2).

#### 3.4.4. Residual Astigmatism

The mean postoperative refractive cylinder was low: −0.52 ± 0.48 D (Table 4), indicating a good toric performance (from mean −2.80 D preoperatively, *n* = 130 with measurable preoperative refraction, to −0.52 D, *n* = 161, achieved postoperatively). A strong clinical performance of cylinder correction is shown in Figure 3G, presenting a total of 62% eyes with residual refractive astigmatism ≤ 0.50 D and 89% ≤ 1.00 D. Eighteen eyes (11.2%) had residual refractive astigmatism exceeding 1.00 D. Factors associated with greater residual astigmatic error are examined in the multivariate regression analysis (Section 3.7). A detailed vector analysis of the astigmatic correction (at the corneal plane, *n* = 161) is presented subsequently.

#### 3.4.5. Defocus Equivalent (DEQ)

**Figure 4:**
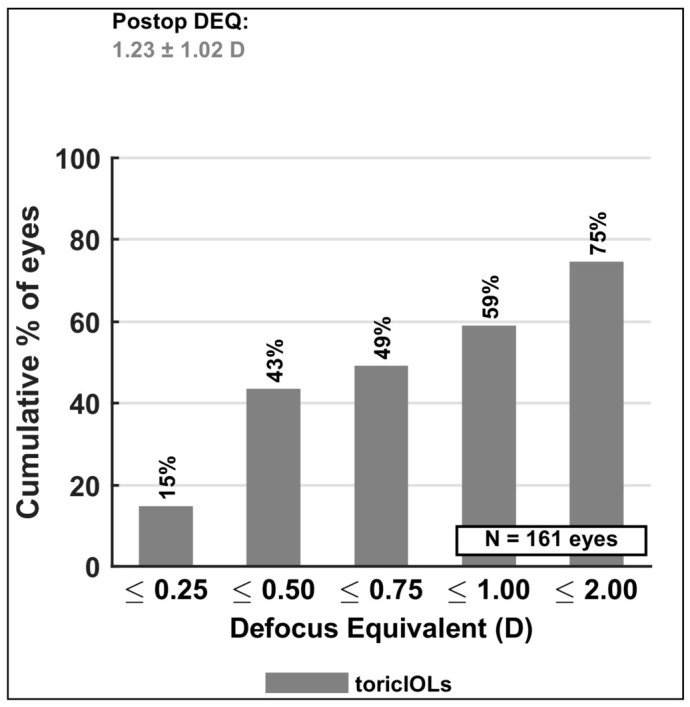
Defocus Equivalent (DEQ) Histogram (DEQ integrates both spherical and cylindrical components of refraction into a single metric assessing refractive blur, *n* = 161, D—Diopters). Generated with mEYEstro version 2.5.0.0 [29].

In confirmation of the SE and residual cylinder interpretation, the DEQ histogram is presented in Figure 4. DEQ, which integrates both the spherical and the cylindrical components of refraction into a single metric assessing refractive blur (Figure 4). The post-operative DEQ was 1.23 ± 1.02 D; 59% of eyes had a DEQ ≤ 1.00 D. The relatively wide distribution of DEQ values compared to cylinder alone reflects the inclusion of eyes targeted for residual myopia, which by design carry a higher spherical component.

### 3.5. Vector Analysis

#### 3.5.1. Vector Analysis of Astigmatism

Vector analysis of astigmatic outcomes according to the Alpins method [23] (presented in Table 5) demonstrated a mean target-induced astigmatism (TIA) of 2.76 ± 1.39 D and a mean surgically induced astigmatism (SIA) of 2.88 ± 1.43 D. The mean difference vector (DV) was 0.53 ± 0.44 D (median 0.42 D), indicating low residual astigmatism. The mean DV closely corresponded to the mean residual refractive cylinder, supporting the internal consistency of the vector analysis. A separate sensitivity analysis presented in Table A2 excludes two patients with intraoperative events that could influence astigmatism readings (posterior capsule rupture, corneal suture). This analysis quantifies exact measurement differences and demonstrates the robustness of vectorial analysis results.

**Figure 5:**
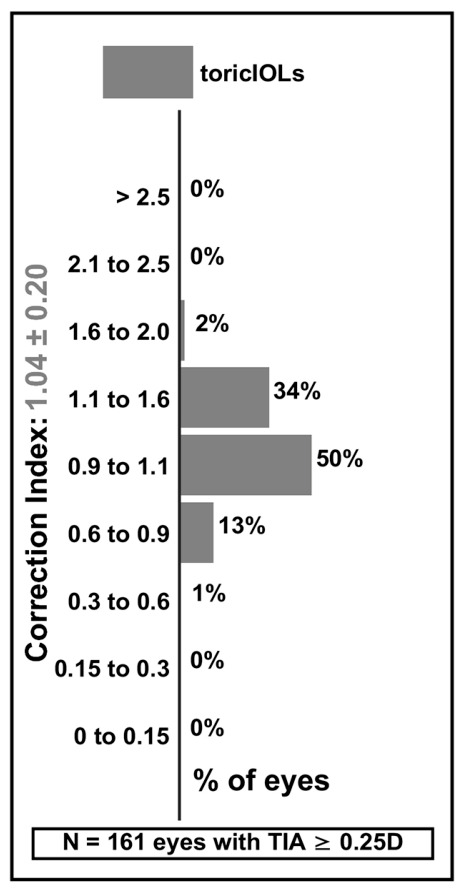
Corrective Index (CI) analysis distribution at the primary endpoint (one month postoperative) was generated using mEYEstro version 2.5.0.0 [29]. D, Diopter; IOL, intraocular lens; *N*, number of (eyes).

The mean correction index (CI) was 1.04 ± 0.20 (Table 5 and Figure 5), suggesting a slight tendency toward overcorrection (a mean overcorrection of 4% at the level of the entire study cohort), with half (50.3%) of eyes within the clinically acceptable target range (CI 0.9–1.1), 36.0% overcorrected (CI > 1.1), and 13.7% undercorrected (CI < 0.9). The correction index was calculated using full vector arithmetic per the Alpins method [23]; the scalar ratio of SIA to TIA magnitudes (scalar approximation) was 1.055, marginally exceeding the vectorial CI of 1.037, with the small difference attributable to minor axis misalignment between the induced and intended corrections. The index of success (IOS) was 0.22 ± 0.18, reflecting the low postoperative astigmatism relative to the magnitude of the intended correction.

The mean angle of error was centered around zero (−0.15° ± 5.37°, median 0°), indicating minimal systematic rotational error (Table 5). A total of 70.8%, 91.9%, and 97.5% of eyes were within ±5°, ±10°, and ±15° of the intended toric axis, respectively. The symmetrical distribution of the AE histogram with tight clustering around 0° indicates that any axis deviations were not systematic and no consistent tendency toward either under- or over-rotation is involved (Figure 3I).

The TIA versus SIA regression demonstrated a slope of 0.97 (R^2^ = 0.89; Figure 3H), confirming strong proportionality between the intended and achieved astigmatic correction across the full range of TIA magnitudes. The R^2^ for the TIA-SIA regression is lower than the 0.99 observed for the SE regression, reflecting the inherently greater variability of astigmatic correction compared to spherical correction alone.

#### 3.5.2. Double-Angle Plot Analysis of Astigmatism

**Figure 6:**
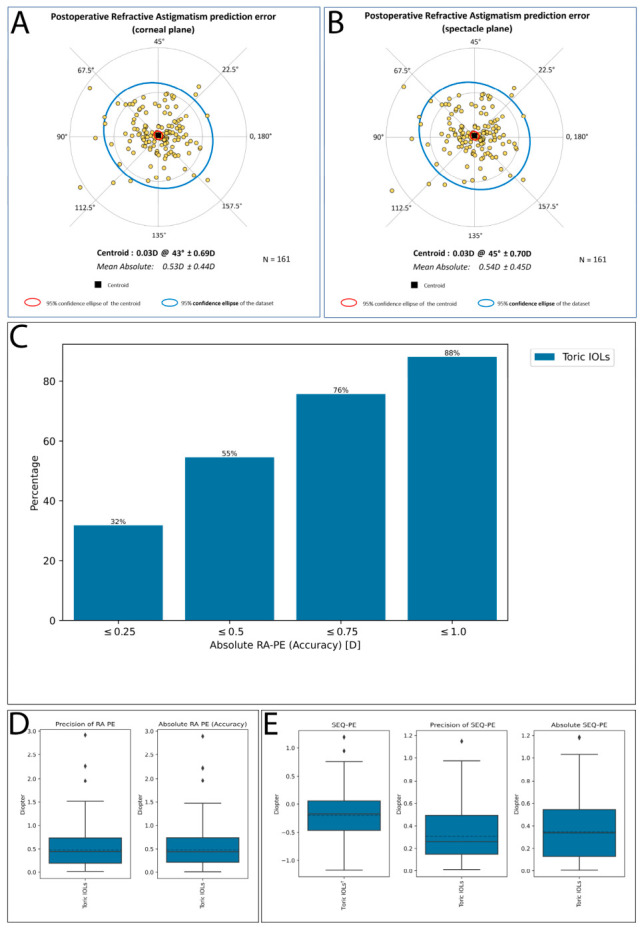
Double-angle plot analysis of postoperative refractive astigmatism prediction error and astigmatic correction accuracy (*n* = 161 eyes). (**A**,**B**) Double-angle plots of the postoperative refractive astigmatism prediction error at the corneal plane (**left**) and spectacle plane (**right**), image generated using the downloaded ASCRS Double-Angle Plot Tool v1.32 (from https://www.ascrs.org/en/tools/astigmatism-double-angle-plot-tool) (accessed on 28 March 2026) [24]. Each data point represents one eye. The black square denotes the centroid (vectorial mean). The red ellipse represents the 95% confidence ellipse of the centroid; the blue ellipse represents the 95% confidence ellipse of the dataset. Ring scale = 1.00 D. (**C**) Cumulative distribution of absolute RA-PE (thresholds: ≤0.25, ≤0.50, ≤0.75, and ≤1.00 D. (**D**) Boxplots of the refractive astigmatism prediction error (RA-PE) magnitude, showing precision and accuracy. (**E**) Spherical Equivalent prediction error (SE-PE); C-E generated using the Eyetemis Analysis Tool (Available online: https://www.eyetemis.com accessed on 24 March 2026, endorsed by ESCRS) [25,26,27,28]; for panels (**D**,**E**): solid line = median; dashed line = mean; diamonds = outliers. All Eyetemis analyses were performed at the spectacle plane (vertex distance 12 mm). RA-PE, refractive astigmatism prediction error; SE-PE, spherical equivalent prediction error; D, diopters; ASCRS, American Society of Cataract and Refractive Surgery.

Double-angle plots at both the spectacle and corneal planes demonstrated a centroid of 0.03 D @ 45° ± 0.70 D and 0.03 D @ 43° ± 0.69 D (Figure 6A,B), respectively, indicating negligible systematic bias in the toric calculator predictions with no consistent directional error. The mean absolute residual astigmatism prediction error was 0.54 ± 0.45 D at the spectacle plane and 0.53 ± 0.44 D at the corneal plane, with close agreement between the two measurement planes, further supporting the robustness of the findings (the vertex distance conversion did not substantially affect the results).

Analysis of the postoperative refractive astigmatism prediction error (RA-PE) is also presented in Figure 6C,D. The distribution of RA-PE values was moderately dispersed, with most observations clustered within ±0.50 D and a limited number of higher-magnitude outliers, as illustrated in both the double-angle plots and boxplots. In terms of accuracy thresholds, 32% of eyes achieved an absolute RA-PE of ≤0.25 D, 55% ≤ 0.50 D, 76% ≤ 0.75 D, and 88% ≤ 1.00 D (Figure 6C). The precision of RA-PE, defined as the distance of each individual observation from the centroid, is illustrated in Figure 6D and shows a median of approximately 0.45 D (IQR 0.2–0.75 D), consistent with the dispersion observed across the dataset.

Vector analysis characterizes both magnitude and orientation of astigmatic error, whereas scalar metrics reflect magnitude alone; together, these findings indicate that residual astigmatism was not directionally biased. Overall, these results suggest that toric IOL calculations provided accurate and unbiased astigmatic correction at the group level, although moderate variability remained at the individual eye level.

### 3.6. Spectacle Independence

At one month postoperatively, complete spectacle independence was achieved in 18 of 118 patients (15.3%) (Table 6), while the remaining 84.7% required some form of optical correction. The most common pattern was dependence on near correction only (46.6%), followed by distance-only prescription (25.4%) and combined distance and near optical correction (11.0%). A small proportion of patients (1.7%) demonstrated discordant spectacle status between eyes and were classified as spectacle dependent.

When analyzed according to refractive strategy, success was defined as achievement of the intended postoperative functional outcome for each group: distance-only spectacle prescription issuance for minus-target monofocal eyes, reading-only spectacle use for emmetropia-target monofocal eyes, and complete spectacle independence for presbyopia-correcting IOL eyes. Defined in this way, the intended functional outcome was achieved in 85.3% of minus-target monofocal cases (*n* = 34), 72.6% of emmetropia-target monofocal cases (*n* = 62), and 63.6% of presbyopia-correcting IOL cases (*n* = 22). The 27.4% of emmetropia-targeted monofocal patients who required distance correction represent cases that did not achieve the intended emmetropic target, consistent with the spherical equivalent prediction error data reported above. The lower rate of complete spectacle independence in the presbyopia-correcting IOL group (63.6%) likely reflects, in part, the sensitivity of these lenses to residual refractive error and should be interpreted in the context of the higher-than-average preoperative corneal astigmatism in this cohort. Although numerically higher success rates were observed in the minus-target group, the overall difference across refractive strategies did not reach statistical significance (χ^2^ test, *p* = 0.167).

When success was defined as achievement of the intended strategy-specific functional outcome, the majority of patients in each refractive group met their predetermined endpoint, with comparable rates across strategies despite the heterogeneity of the cohort.

### 3.7. Multivariate Analysis: Predictors of Residual Astigmatic Error

To identify independent predictors of residual astigmatic error, a multiple linear regression analysis was performed with the difference vector (DV) magnitude as the outcome variable. Candidate predictors included preoperative corneal astigmatism magnitude, axial length, age, IOL cylinder power, CTR use, surgery type, astigmatism orientation, and intended refractive target and were first screened by univariate linear regression (Table A1). Variables achieving *p* < 0.20 were entered into the multivariate model: IOL cylinder power (univariate *p* = 0.003), CTR use (*p* = 0.162), and astigmatism orientation (categorical, *p* = 0.041). All remaining variables showed no significant univariate association with DV magnitude (all *p* > 0.32), including preoperative corneal astigmatism magnitude, axial length, age, and intended refractive target.

In the multivariate model (Table 7), IOL cylinder power was a significant independent predictor of DV magnitude (β = 0.051, 95% confidence interval 0.005–0.096, *p* = 0.031), with each additional diopter of IOL cylinder power associated with a 0.051 D increase in residual astigmatic error. As a non-parametric sensitivity analysis, Spearman rank correlation between IOL cylinder power and DV magnitude yielded a borderline association (r_s_ = 0.139, *p* = 0.080) that may reflect the limited resolution introduced by the discrete stepwise increments of available toric IOL cylinder powers. Taken together, these findings suggest a weak but consistent association between higher IOL cylinder power and greater residual astigmatic error.

Oblique astigmatism orientation was independently associated with higher DV magnitude compared to with-the-rule astigmatism (β = 0.299, 95% confidence interval 0.027–0.570, *p* = 0.032). Mean DV magnitude was 0.83 ± 0.79 D in oblique eyes versus 0.49 ± 0.40 D in WTR eyes and 0.53 ± 0.38 D in ATR eyes, with no significant difference between WTR and ATR (*p* = 0.343). This finding suggests that toric correction was less predictable in oblique astigmatism orientation in this cohort, potentially reflecting greater variability in toric calculator accuracy or axis alignment at non-horizontal and non-vertical meridians. This finding should be interpreted cautiously, given the small number of oblique astigmatism cases (*n* = 12).

CTR use did not reach significance after adjustment (β = 0.145, *p* = 0.130), with the positive direction of the coefficient likely reflecting confounding by case profile and complexity rather than a true adverse effect of CTR on astigmatic outcomes, as CTRs were selectively used in eyes with high myopic pre-operative refractive status or zonular weakness or other complicating factors that may independently affect toric alignment precision.

Regression assumption diagnostics indicated mild right skew of residuals and mild heteroscedasticity, consistent with the bounded non-negative nature of the DV magnitude outcome. No clear pattern suggesting model misspecification was observed. Given the sample size of 161 eyes, these deviations are not expected to substantially affect the validity of the findings. Additionally, inclusion of bilateral cases (36.4% of patients) introduces a partial breach of the independence assumption, and results should be interpreted with this limitation taken into account.

### 3.8. Stability Sub-Analysis

A subset of 75 eyes (46.6% of the total cohort) had extended follow-up data available beyond the primary 1-month endpoint, with a mean follow-up of eight months (median 7 months, range 2–25 months). The distribution of follow-up duration was as follows: 44.0% between 2 and 6 months, 40.0% between 7 and 12 months, 8.0% between 13 and 18 months, and 6.7% beyond 18 months (Table 8).

Refractive stability is illustrated in Figure 3F. Mean SE in this subset was −0.71 ± 1.24 D at one month and −0.68 ± 1.22 D at the last available follow-up visit (median seven months), corresponding to a mean change of +0.02 ± 0.31 D, indicating a negligible hyperopic shift without clinical significance. A total of 90.7% of eyes demonstrated SE change within ±0.50 D between the 1-month and the latest follow-up available, with only 9.3% showing a change exceeding 0.50 D.

These findings indicate stable refractive outcomes beyond the primary endpoint. As this subset was not a predefined cohort but reflects eyes with clinically driven return visits, these results should be interpreted with appropriate caution regarding selection bias.

### 3.9. Subanalysis: Emmetropia-Targeted vs. Minus-Targeted Monofocal IOL Eyes

The study cohort included a substantial proportion of eyes specifically targeted for postoperative myopia (29.8%), in which preserving uncorrected postoperative near vision was considered preferable to inducing spectacle dependence for near tasks in previous high myopic patients. To evaluate whether the intended spherical refractive strategy influenced astigmatic correction accuracy, outcomes were compared between monofocal emmetropia-targeted eyes (*n* = 78) and monofocal minus-targeted eyes (*n* = 48) (Table 9). Presbyopia-correcting toric IOL eyes were excluded from this comparison, given their fundamentally different optical design and functional goals.

Astigmatic correction accuracy was comparable between groups across all primary vector metrics. DV magnitude did not differ significantly between emmetropia-targeted and minus-targeted eyes (median 0.447 D vs. 0.348 D, *p* = 0.310), nor did residual cylinder (median 0.50 D vs. 0.38 D, *p* = 0.370), angle of error (mean 0.02° vs. 0.19°, *p* = 0.858), or the proportion of eyes achieving residual cylinder ≤ 0.50 D (66.7% vs. 70.8%, *p* = 0.771) or ≤1.00 D (91.0% vs. 91.7%, *p* = 1.000). These findings indicate that toric IOL astigmatic accuracy was equivalent regardless of the intended spherical target.

The correction index showed a statistically significant difference between groups (median 1.019 vs. 0.975, *p* = 0.049), with emmetropia-targeted eyes demonstrating marginally greater correction relative to the target. While this difference reached statistical significance, both values remain within the clinically acceptable range close to the ideal value of 1.0, and the magnitude of difference (0.044) is unlikely to be clinically meaningful. This finding may partly reflect systematic differences in IOL effective lens position between emmetropic and myopic eyes.

Spherical equivalent prediction error was similar between groups (median −0.185 D vs. −0.165 D, *p* = 0.746), indicative of equivalent spherical accuracy of the IOL power calculations across both sub-groups. Postoperative CDVA was also comparable, with both groups achieving a median of 0.04 logMAR (approximately 20/22), without a statistically significant difference (*p* = 0.077).

### 3.10. Cataract Surgery vs. Refractive Lens Exchange Subgroup Analysis

Because cataract surgery and refractive lens exchange (RLE) represent clinically distinct populations, a subgroup comparison was performed. To evaluate whether surgical indication influenced outcomes, primary visual, refractive, and astigmatic vector metrics were compared between cataract surgery (*n* = 133) and refractive lens exchange (*n* = 28) subgroups (Table 10).

Postoperative UDVA and CDVA were significantly better in RLE eyes (UDVA: median 0.00 vs. 0.10 logMAR, *p* = 0.043; CDVA: median 0.00 vs. 0.05 logMAR, *p* = 0.031), likely reflecting differences in baseline ocular status and surgery indications, with lower prevalence of visual acuity-limiting comorbidities, and greater visual potential in the RLE subgroup rather than superior refractive correction.

In contrast, all astigmatic correction and refractive accuracy metrics were comparable between groups. Difference vector magnitude (median 0.40 vs. 0.47 D, *p* = 0.646), correction index (median 1.02 vs. 0.97, *p* = 0.254), index of success (median 0.18 in both groups, *p* = 0.805), residual cylinder (median 0.47 vs. 0.50 D, *p* = 0.504), spherical equivalent prediction error (median −0.18 vs. −0.20 D, *p* = 0.836), and the proportion of eyes achieving residual cylinder ≤ 0.50 D (60.9% vs. 67.9%, *p* = 0.529) and ≤1.00 D (88.0% vs. 92.9%, *p* = 0.741) did not differ significantly between groups. Angle of error also did not differ significantly (*p* = 0.095), indicating similar rotational accuracy across surgical indications.

These findings indicate that toric IOL astigmatic correction accuracy was equivalent across both surgical indications, with visual acuity differences clinically attributable to baseline characteristics rather than refractive performance.

### 3.11. Postoperative Complications and Intraoperative Events

Intraoperative and postoperative complications are summarized in Table 11. One intraoperative complication occurred: a posterior capsule rupture requiring anterior vitrectomy (0.6%). A corneal suture was placed at the main incision in one case (0.6%) for wound security; this was not considered a complication but an intraoperative safety measure. Additional planned concomitant procedures included two intravitreal anti-VEGF injections for pre-existing macular pathology (1.2%), one pupilloplasty in a case of traumatic cataract with iris irregularity (0.6%), and one synechiolysis between the iris and anterior capsule in an eye with sequelae of prior uveitis (0.6%).

In the immediate postoperative period, transient corneal edema was observed in 10 eyes (6.2%), resolving completely in all cases prior to the 1-month assessment. Intraocular pressure elevation was recorded in four eyes (2.5%), all occurring in the eyes without pre-existing glaucoma and resolving with topical anti-glaucoma medication and adjustment of the postoperative steroid taper. Dry eye and ocular surface complaints were reported in three eyes (1.9%), and one case of conjunctivitis (0.6%) resolved without sequelae.

Toric IOL rotation of ≥15° was identified on slit-lamp evaluation and documented in 2 eyes (1.2%): one involving an AcrySof Toric IQ T5 implanted in a high myopia eye (AL of 29.8 mm, no CTR), targeted for postoperative myopia, and one in a Miniwell Toric case with CTR. Residual refractive astigmatism was 0.75 D in both eyes, and functional visual outcomes were satisfactory, precluding the need for surgical repositioning. One case required distance optical correction in line with the intended refractive strategy, while the other did not require spectacle correction. Both eyes were managed conservatively with observation.

Posterior capsule opacification was detected in seven eyes (4.3%) during the primary follow-up period, of which four underwent Nd:YAG capsulotomy (2.5%).

No cases of endophthalmitis, cystoid macular edema, retinal detachment, or permanent vision loss were recorded.

A sensitivity analysis excluding the two eyes with intraoperative events (posterior capsule rupture and corneal suture; *n* = 159) demonstrated no meaningful change in refractive or vector outcomes (Table A2). Residual cylinder ≤ 0.50 D and ≤1.00 D was achieved in 66.0% and 92.5% of eyes, respectively, and vector metrics (DV, CI, IOS) remained within similar ranges to the primary analysis. The angle of error remained centered around zero (mean −0.05° ± 5.11°, median 0.00°), confirming the absence of systematic rotational bias.

## 4. Discussion

Several studies have described in depth astigmatism correction via toric intraocular lenses in homogeneous case–controlled cohorts. This study aims to compare previously described literature findings versus a heterogeneous 161-eye cohort, which could be more relevant to day-to-day clinical practice. Thus, the study evaluated the real-world visual, refractive, and astigmatic outcomes of toric IOL implantation in a consecutive single-surgeon cohort of 161 eyes with preoperative corneal astigmatism ≥ 2.00 D, encompassing both cataract surgery and refractive lens exchange across a broad spectrum of IOL optical designs and refractive strategies. Eyes with prior corneal refractive surgery were excluded due to their distinct corneal biomechanics and IOL calculation considerations (dedicated studies are warranted to evaluate toric IOL outcomes in this subgroup).

Statistical analysis was performed firstly for the entire study cohort and secondly for the populational subgroups (such as refractive lens exchange patients versus cataract patients, patients with preoperative visual comorbidities versus those without). The purpose of the secondary analyses of populational groups was multifold: to verify if heterogeneity of the cohort could impact findings by assessing if populational subgroups differ significantly from the entire cohort, and to search for additional relevant clinical correlations.

The principal findings are as follows: toric IOL implantation achieved substantial and predictable correction of both spherical and astigmatic refractive error, with a mean residual cylinder of 0.52 D, a correction index of 1.04, near-zero centroid deviation on double-angle plot analysis, and 88.8% of eyes within ±1.00 D of residual astigmatism. Refractive stability was confirmed in the extended follow-up subgroup. Two independent predictors of residual astigmatic error were identified: IOL cylinder power (consistent with the established role of preoperative astigmatism magnitude as the principal determinant of residual error) and oblique astigmatism orientation, a finding consistent with and supported by the published literature on the incomplete predictability of this astigmatism type. A dedicated sub-analysis demonstrated that toric IOL astigmatic correction accuracy was not materially affected by the intended spherical refractive target, supporting the use of toric IOLs in highly myopic patients targeted for intentional residual myopia. The safety profile was favorable, with no sight-threatening complications and an 88.2% complication-free rate at the primary endpoint.

### 4.1. Visual, Refractive, and Vector Analysis Outcomes

Visual, refractive, and vector outcomes in this cohort demonstrate a high level of overall surgical performance, despite the inclusion of eyes with substantial preoperative astigmatism (≥2.00 D) and a heterogeneous real-world case mix of IOL models, intended refractive targets and surgery types (cataract and RLE).

Toric IOL implantation achieved substantial visual rehabilitation across the entire cohort following surgery. Mean UDVA improved from 1.29 ± 0.67 to 0.19 ± 0.24 logMAR and mean CDVA from 0.55 ± 0.51 to 0.09 ± 0.15 logMAR, with 37% and 48% of eyes achieving UDVA and CDVA of 20/20 or better, respectively. These visual outcomes should be interpreted in context: preoperative CDVA in this cohort was frequently limited by advanced lens opacities rather than refractive error alone, resulting in safety and efficacy indices that reflect substantial visual rehabilitation rather than purely refractive performance. No eye experienced a loss of two or more lines of CDVA. These findings are consistent with the evidence base from randomized controlled trials demonstrating that toric IOLs provide superior uncorrected distance visual acuity and lower residual astigmatism compared with non-toric IOLs, limbal relaxing incisions [31,32], and the newer femtosecond laser-assisted astigmatic keratotomy (FSAK) [12]. Importantly, the gap between UDVA and CDVA distributions in the present cohort is partly attributable to the inclusion of 29.8% of eyes intentionally targeted for residual myopia, in whom uncorrected distance acuity was not the primary functional endpoint and should not be interpreted as a marker of suboptimal correction. Furthermore, in the subgroup of eyes without preoperatively documented relevant visual comorbidities (*n* = 126), mean postoperative CDVA was 0.03 ± 0.05 logMAR (median 0.00), confirming that the visual acuity outcomes in the primary cohort are not indicative of suboptimal refractive performance but rather also reflect the real-world inclusion of eyes with concurrent ocular pathology.

Spherical equivalent refractive accuracy relative to postoperative target was high: 69.6% of eyes were within ± 0.50 D and 98.1% within ± 1.00 D of the intended spherical target, with a mean prediction error of −0.19 ± 0.42 D indicating a slight overall myopic shift. This mild myopic trend might be consistent with the known tendency of several IOL calculation formulas to produce a relative myopic shift in long eyes [33], which comprised 23.6% of the present cohort, and is of modest clinical magnitude. The attempted versus achieved spherical equivalent regression demonstrated excellent linearity (slope 1.01, R^2^ = 0.99), confirming consistent spherical predictability across the full range of attempted corrections.

Astigmatic outcomes were similarly effective, with a mean postoperative refractive cylinder being 0.52 ± 0.49 D, a substantial reduction from a mean preoperative corneal cylinder of 2.76 D, with 62% of eyes achieving ≤ 0.50 D and 88.8% ≤ 1.00 D residual cylinder. Qiu et al. reported satisfactory astigmatic correction in a cataract cohort with corneal astigmatism ≥ 2.57 D [34], confirming that contemporary toric IOL platforms and calculators perform reliably in high-astigmatism eyes.

Vector analysis further characterizes the precision of astigmatic correction. The mean DV of 0.53 ± 0.44 D closely corresponded to the mean residual refractive cylinder, confirming internal consistency between scalar and vector measures. The CI of 1.04 ± 0.20 indicated a slight tendency toward overcorrection at the population level, with 50.3% of eyes within the target range (CI 0.9–1.1), 36.0% mildly overcorrected, and 13.7% undercorrected. The CI outcome is comparable with the value of 1.04 ± 0.35 reported in a large toric multifocal IOL series [35], supporting the robustness of this metric across different IOL platforms and surgical indications. The positive intercept of the TIA versus SIA regression (y = 0.97x + 0.20, R^2^ = 0.89) is consistent with this pattern, suggesting the overcorrection tendency is more pronounced at lower cylinder magnitudes. The mean AE was centered around zero (−0.15° ± 5.37°), with 70.8% and 91.9% of eyes within ±5° and ±10° of the intended axis, respectively, indicating accurate toric alignment without systematic rotational bias. These rotational outcomes compare favorably with the pooled mean absolute postoperative rotation of 2.36° (95% confidence interval 2.08–2.64°) reported across 51 published studies and 4863 eyes in a recent meta-analysis of toric IOL rotational stability [13]. Double-angle plot analysis demonstrated near-zero centroid deviation at both the spectacle plane (0.03 D@45°) and corneal plane (0.03 D@43°), confirming the absence of systematic directional prediction error across the cohort—a key quality indicator recommended by current reporting standards [24,26].

Taken together, the scalar, vector, and graphical analyses demonstrate consistent, accurate, and well-calibrated toric IOL performance across a heterogeneous high-astigmatism real-world cohort, with results meeting contemporary benchmarks for both spherical and astigmatic correction.

### 4.2. Spectacle Dependence and Achievement of Intended Functional Outcome

Spectacle dependence status was assessed at the patient level at one month postoperatively and analyzed both as an overall distribution and as a strategy-specific functional outcome metric. Complete spectacle independence was achieved in 15.3% of patients (18/118). This figure should not be interpreted in isolation: the present cohort was predominantly composed of monofocal IOL recipients (78.9% of eyes), a proportion of whom were intentionally targeted for residual myopia to preserve near function, and for whom uncorrected distance independence was neither the surgical goal nor a meaningful outcome metric. In this context, the overall spectacle independence rate primarily reflects cohort composition rather than a deficit in refractive performance.

A strategy-specific success analysis was performed to provide a more clinically meaningful assessment of functional outcomes than global spectacle independence alone. Achievement of the intended functional outcome was defined separately for each refractive strategy: distance-only spectacle use for minus-targeted monofocal eyes, reading-only spectacle use for emmetropia-targeted monofocal eyes, and complete spectacle independence for presbyopia-correcting IOL eyes. Defined in this way, the intended outcome was achieved in 85.3% of minus-targeted eyes, 72.6% of emmetropia-targeted monofocal eyes, and 63.6% of presbyopia-correcting IOL eyes, with no statistically significant difference across groups (*p* = 0.167). This framework of evaluating success relative to the intended refractive strategy rather than against a universal benchmark is particularly relevant in heterogeneous real-world cohorts and avoids penalizing intentional non-emmetropic outcomes as failures. To our knowledge, this strategy-specific success metric in a mixed toric IOL cohort has been rarely reported, and we consider it a clinically meaningful addition to standard spectacle independence reporting.

The success rate in the presbyopia-correcting IOL subgroup (63.6%, *n* = 22) was numerically lower than in the monofocal groups and below rates reported in dedicated toric presbyopia-correcting IOL series. For example, other real-world studies of toric EDOF and trifocal IOLs, including those by Nguyen et al. in a Vivity toric EDOF [36] and the Canadian PanOptix [35,37] cohorts, provide a broadly comparable clinical context, although direct comparison is limited by differences in baseline astigmatism, cohort composition, and definitions of spectacle dependence (the present analysis was based on clinically prescribed correction, not patient-reported use of glasses, in a cohort with higher preoperative astigmatism). Mohaseb et al. [37] reported that 79% of patients implanted with PanOptix Toric IOL rarely or never required near spectacles, and 96% achieved distance independence. This difference likely reflects the greater sensitivity of presbyopia-correcting IOLs to residual refractive error, where even small amounts of residual astigmatism may compromise multifocal or extended-range optical performance and reduce functional spectacle independence [31]. In addition, the relatively small size of this subgroup (*n* = 22) limits the precision of the estimate, with a wide confidence interval around the observed success rate.

Taken together, the spectacle dependence data confirm that toric IOL implantation achieved its intended functional goals across all three refractive strategies, with overall patterns consistent with the published literature when interpreted within the appropriate clinical context of each analyzed subgroup.

### 4.3. Emmetropia vs. Intentional Myopia (Minus-Target) Sub-Analysis

A clinically relevant subgroup in our cohort consisted of highly myopic patients in whom intentional residual myopia was targeted instead of emmetropia. This strategy aims to preserve uncorrected near vision, which these patients have relied on lifelong, while simultaneously correcting corneal astigmatism to optimize visual quality at the intended focal distance. In this setting, toric IOL implantation serves a dual purpose: accurate astigmatic correction combined with a planned myopic offset to maintain functional near vision without spectacles.

We therefore performed a dedicated sub-analysis comparing toric monofocal IOL outcomes between eyes targeted for emmetropia (*n* = 78) and those targeted for residual myopia (*n* = 48, with the majority targeted for −2.50 D), to evaluate whether the intended spherical refractive target influences astigmatic correction performance.

Published data addressing this question is limited. Shin et al. demonstrated that toric IOLs improve refractive astigmatism and visual outcomes compared with monofocal IOLs in eyes targeted for myopia [38], supporting their use even when emmetropia is not the intended endpoint [38]. However, that study did not compare astigmatic accuracy between different refractive targets [38]. Sakai et al. reported reduced accuracy in spherical equivalent prediction when targeting intentional myopia, with a tendency toward myopic shift [33,39], suggesting that refractive target may influence overall IOL calculation performance [33,39] though this effect may be attenuated with newer generation formulas, and its impact on toric outcomes remains unaddressed. Guo et al. compared toric IOL outcomes directly between high myopia eyes targeted for approximately −3.00 D and emmetropia/low myopia eyes [40], finding no significant difference in residual astigmatism or rotational stability between groups—the closest published comparator to our sub-analysis in terms of both IOL type and the magnitude of intended myopic offset [40].

In the present study, astigmatic correction outcomes were comparable between groups across all primary metrics. Residual cylinder (0.500 vs. 0.375 D, *p* = 0.370), the proportion of eyes achieving ≤ 0.50 D and ≤1.00 D of residual astigmatism (66.7% vs. 70.8% within 0.50 D residual cylinder, *p* = 0.771), angle of error (0.02° vs. 0.19°, *p* = 0.858), and DV magnitude (0.447 vs. 0.348 D, *p* = 0.310) did not differ significantly between emmetropia- and minus-targeted eyes, indicating that toric IOL astigmatic performance was not materially affected by the intended spherical refractive target. Postoperative CDVA was also equivalent between groups (0.04 logMAR in both, *p* = 0.077), suggesting that optical quality was preserved with the myopic targeting strategy.

The correction index was the only parameter reaching borderline statistical significance, with a slightly higher value in emmetropia-targeted eyes (1.019 vs. 0.975, *p* = 0.049).

However, both values fall within the clinically acceptable range (0.9–1.1), and the small absolute difference of 0.044 is unlikely to be clinically meaningful. This finding may reflect some differences in effective lens position or IOL power calculations in myopic eyes, although this remains speculative within our analysis.

These findings are clinically relevant for the management of highly myopic patients with significant corneal astigmatism, in whom surgeons must balance accurate astigmatic correction with preservation of functional near vision. Our results suggest that these goals are not in conflict, and that toric IOL astigmatic accuracy appears robust across the different refractive strategies employed. Further studies with standardized Alpins methodology [23] are warranted to confirm these findings and to explore the underlying mechanisms of the small differences observed, particularly in cohorts of highly myopic eyes undergoing toric IOL implantation with intentional myopic targets, where this question is most clinically relevant.

### 4.4. Predictors of Residual Astigmatic Error—Multivariate Regression

Multivariate linear regression identified two independent predictors of residual astigmatic error (DV magnitude): IOL cylinder power and oblique astigmatism orientation. The overall model explained a modest but statistically significant proportion of variance (R^2^ = 0.089, adjusted R^2^ = 0.066, *p* = 0.005), indicating that a proportion of variability remains unexplained, consistent with the multifactorial nature of toric IOL outcomes.

The association between higher IOL cylinder power and greater residual astigmatic error (β = 0.051 per diopter, *p* = 0.031) should be considered consistent with established literature and with toric IOL optics. Preoperative corneal astigmatism magnitude has been identified as the principal predictor of residual postoperative astigmatism in multiple series [41,42], and IOL cylinder power—being largely determined by preoperative corneal astigmatism — may function as a proxy for the magnitude of attempted correction. Additionally, each degree of rotational error reduces astigmatic correction by approximately 3.3% [14,15], so that any residual calculational or alignment imprecision translates into a proportionally larger DV at higher cylinder powers. The borderline Spearman correlation (r_s_ = 0.139, *p* = 0.080) likely reflects the limited statistical resolution imposed by the discrete fixed increments of available toric IOL cylinder powers rather than a true absence of association, and both the parametric and non-parametric analyses taken together support a weak but consistent relationship. Although preoperative astigmatism magnitude has been identified as a predictor of residual astigmatism in prior studies [41,42], in our present analysis, it did not reach the screening threshold. This may reflect the use of DV magnitude instead of scalar residual cylinder as the outcome variable, as well as the restricted inclusion of eyes with high baseline astigmatism (≥2.00 D).

The more clinically impactful finding was the independent association between oblique astigmatism orientation and higher residual astigmatic error compared with WTR (β = 0.299, 95% confidence interval 0.027–0.570, *p* = 0.032). Mean DV was higher in oblique eyes (0.83 ± 0.79 D) than in WTR (0.49 ± 0.40 D) and ATR eyes (0.53 ± 0.38 D), whereas ATR orientation was not a significant predictor relative to WTR, suggesting that in this cohort, toric correction was less predictable in oblique astigmatism.

This finding is consistent with prior literature. Several studies have highlighted clinical difficulty in correctly calculating toric IOL correction for oblique astigmatism [4,43,44]. A prospective multisite study of 218 eyes by Ninomiya Y. et al. [4] showed that the centroid error in predicted residual astigmatism was close to the origin in WTR eyes but shifted significantly in both ATR and oblique groups when planning was based on anterior corneal curvature alone, with oblique eyes performing comparably to ATR in terms of correction inaccuracy [4]. The mechanistic basis lies in the axis-dependent contribution of posterior corneal astigmatism; Koch et al. established that ignoring posterior corneal astigmatism introduces systematic prediction errors that differ in direction and magnitude by axis orientation [43], with WTR eyes prone to overcorrection and ATR eyes to undercorrection [43]. Extending the issue specifically to oblique astigmatism, Nakano S. et al. found that, unlike WTR and ATR eyes, incorporating direct posterior corneal measurements via AS-OCT did not significantly improve toric IOL outcomes in oblique eyes [44]. At a broader level, Kawahara et al. confirmed that while WTR and ATR components are now more reliably predicted, the oblique component remains less predictable, with prediction still considered incomplete [41,42].

The oblique subgroup comprised only 12 eyes (12/161), and this finding should therefore be interpreted cautiously. The wide confidence interval (0.027–0.570) and elevated DV standard deviation in oblique eyes reflect this limited sample size, and the result may be influenced by individual outliers. Nonetheless, its consistency with published literature supports its biological plausibility. We consider this a hypothesis-generating result that warrants confirmation in larger cohorts. As described by Nakano S. et al., oblique astigmatism correction has benefited from the utilization of more accurate anterior segment swept-source OCT Biometers [44] such as the Anterion^®^ (Heidelberg Engineering, Heidelberg, Germany), Argos^®^ (Alcon Healthcare, Fort Worth, TX, USA) [45] and Zeiss IOLMaster 700 (Carl Zeiss Meditec AG, Jena, Germany) systems [46,47]. This study utilized Anterion^®^ for the majority of patients, with some measured using Argos^®^. Even so, these advancements are still insufficient to completely close the gap versus WTR and ATR astigmatism calculation precision. As noted by other larger studies [43,44], the presence of oblique astigmatism may justify greater attention to posterior corneal astigmatism modeling in future toric IOL calculators, particularly those with high preoperative cylinder magnitude.

Expanding further on the subject of oblique astigmatism, some minute differences between biometry devices are expected. Langenbucher A. et al. described finding a lower statistical weight in the oblique vector components in 45°/135°, especially for the corneal back surface power vector, in the Anterion^®^ system versus IOLMaster 700 [47]. Furthermore, the calculated intraocular lens power (IOLP) from Anterion was found systematically higher than that of IOLMaster 700 [47]. Park H. et al. found statistically significant differences with flatter keratometry (K values) readings for Anterion^®^ versus those from Argos^®^ [45]. Song M.Y. et al. Also found flatter keratometry (K values) reading for Anterion^®^ compared to IOLMaster 500 and 700 [48]. Park H. et al. attributed measurement differences to hardware characteristics of the devices [45]. According to Park H. et al., since Argos utilizes data from just 16 data points from projected light-emitting diode (LED) lights on a smaller 2.2 mm zone versus 65 data points from radial scans across a larger 3 mm zone from Anterion, a larger measurement diameter is likely to yield a lower corneal power due to the aspheric and predominantly prolate corneal shape [45]. This mechanistic variance contributes to flatter keratometry measurements [45]. In our study, the largest proportion of patients were measured with Anterion^®^, and some were measured using Argos^®^. Even considering equipment intervariability, our study’s findings on the independent association between oblique astigmatism orientation and higher residual astigmatic error are still consistent with the described literature as previously discussed [43,44].

### 4.5. Safety Profile

The safety profile of the present study was favorable, with low rates of transient postoperative events and no sight-threatening complications. A single intraoperative complication occurred (posterior capsule rupture requiring anterior vitrectomy in one eye), with successful implantation of the planned toric IOL in the capsular bag. In the postoperative period, transient corneal edema (6.2%) and intraocular pressure elevation (2.5%) resolved completely with standard management prior to the 1-month assessment, and no eye experienced a loss of two or more lines of CDVA. Although toric IOL rotation ≥ 15° was identified in two eyes (1.2%), consistent with the low clinically significant rotation rates reported across contemporary toric IOL series [13], neither case required surgical repositioning as functional visual outcomes remained satisfactory. This highlights that the clinical impact of rotational misalignment depends on residual refractive error in the context of patient-specific visual demands and functional tolerance, rather than on rotational magnitude alone, and that conservative management may be appropriate when residual astigmatism falls within functional limits. One of these cases occurred in a highly myopic eye (axial length 29.8 mm) without capsular tension ring implantation, consistent with the known association between increased axial length and rotational instability [17,18,19], further supporting the role of prophylactic CTR use in selected cases. Posterior capsule opacification was detected in 7 eyes (4.3%) during the follow-up period, of which four underwent Nd:YAG capsulotomy (2.5%). No cases of endophthalmitis, cystoid macular edema, retinal detachment, or permanent vision loss occurred. Overall, 88.2% of eyes were free of any complication at the 1-month primary endpoint, confirming an acceptable safety profile consistent with contemporary toric IOL literature in both cataract and refractive lens exchange settings [31,49]. In order to further confirm the robustness of the primary vector outcomes in the context of intraoperative events, a sensitivity analysis excluding the eye with posterior capsule rupture and the eye with prophylactic corneal suture at the main incision was conducted (*n* = 159) and yielded consistent results.

### 4.6. Interpretation of Results from Stability Sub-Analysis

In the subgroup of 75 eyes with extended follow-up (median seven months, range 2–25 months), refractive outcomes remained stable beyond the primary 1-month endpoint. The mean change in spherical equivalent between the 1-month visit and the last available follow-up was +0.02 ± 0.31 D, indicating a negligible and clinically insignificant hyperopic drift. A total of 90.7% of eyes demonstrated a SE change within ±0.50 D, confirming the absence of systematic refractive regression. These findings suggest that the refractive and astigmatic correction achieved at one month is maintained in the short-to-medium term, consistent with the known behavior of modern toric IOLs once rotational stability within the capsular bag is achieved [50]. As this subgroup was not randomly selected but reflects eyes with clinically driven return visits, these results should be interpreted with caution regarding potential selection bias. Nonetheless, the absence of systematic drift across a follow-up range extending to 25 months supports the reliability of the 1-month primary outcome as representative of longer-term refractive performance in this cohort.

### 4.7. Surgical Indication Subgroup Analysis and Cohort Heterogeneity

The heterogeneity of the present cohort, encompassing both cataract surgery and refractive lens exchange (RLE) cases as well as monofocal and presbyopia-correcting toric IOL designs, warrants consideration when interpreting generalizability. Regarding surgical indication, subgroup analysis demonstrated comparable astigmatic correction accuracy between cataract and RLE eyes across all primary vector metrics, despite differences in postoperative visual acuity (UDVA *p* = 0.043; CDVA *p* = 0.031), which are best explained by differences in baseline ocular status, indication for surgery, and visual potential rather than by differences in refractive performance. These findings support the generalizability of the vector analysis results across both surgical indications, while acknowledging that patient expectations and tolerance for residual refractive error differ between these populations.

Regarding IOL optical design, inclusion of both monofocal and presbyopia-correcting toric IOLs introduces optical heterogeneity that may influence visual and functional outcomes, particularly uncorrected visual acuity and spectacle independence, given the differing optical principles of these lenses. Presbyopia-correcting IOLs are known to be more sensitive to small residual refractive errors, including low levels of residual astigmatism, which may impact functional outcomes such as spectacle independence [51,52]. This is reflected in the lower strategy-specific success rate observed in this subgroup (63.6%) compared with monofocal recipients.

However, the primary focus of the present study was the accuracy of astigmatic correction, assessed using vector analysis and refractive metrics, which are largely independent of IOL optical design. In this context, inclusion of multiple IOL types does not materially affect the validity of astigmatism outcome assessment and reflects real-world clinical practice. Nevertheless, this heterogeneity represents a limitation, as it precludes direct comparisons between IOL designs or device-specific conclusions.

### 4.8. Study Strengths and Limitations

The present study has several strengths. It represents a large, single-surgeon, single-center real-world series of toric IOL implantations specifically in eyes with high corneal astigmatism (≥2.00 D), a population underrepresented in the published literature relative to moderate astigmatism cohorts. The inclusion of both cataract and refractive lens exchange cases, multiple IOL optical designs, and a broad range of refractive strategies reflects genuine clinical practice and enhances the external validity of the findings. The application of standardized Alpins vector analysis [23] with cross-validation across multiple software platforms, combined with double-angle plot reporting in accordance with current standards [24,25,26], ensures methodological rigor and comparability with contemporary publications. The dedicated sub-analyses—comparing astigmatic correction accuracy between emmetropia-targeted and intentional myopia-targeted eyes, and identifying oblique astigmatism as an independent predictor of residual error—represent novel contributions to the literature on toric IOL outcomes in complex real-world populations. Clinically, these findings support the use of toric IOLs as a safe, effective, and predictable approach to the simultaneous correction of high corneal astigmatism and its associated spherical ametropia across a wide spectrum of lens surgery indications, including in highly myopic patients for whom preservation of uncorrected near vision is a surgical priority. The data further suggests that toric IOL astigmatic accuracy is not materially affected by the intended spherical refractive strategy, which has direct relevance to surgical planning and patient counseling in this population. Several limitations should be acknowledged. The retrospective analysis of prospectively collected data introduces the possibility of selection bias, and the absence of randomization limits causal inference. The heterogeneity of IOL models and optical designs, while reflecting real-world practice, prevents device-specific conclusions. Extended follow-up data were available for only 46.6% of eyes and were not randomly distributed across the cohort, limiting the power of the stability sub-analysis. The oblique astigmatism subgroup was small (*n* = 12), and this finding should be regarded as hypothesis-generating pending confirmation in larger dedicated series. Finally, systematic IOL rotation data were not available for all eyes.

## 5. Conclusions

In conclusion, this real-world single-surgeon series demonstrates that toric IOL implantation is a safe, effective, and predictable modality for the correction of high corneal astigmatism across a wide range of lens surgery indications, IOL optical designs, and spherical refractive strategies. Astigmatic correction accuracy met contemporary benchmarks across all analytical frameworks—scalar, vector, and graphical—and was maintained over medium-term follow-up. The identification of oblique astigmatism orientation as an independent predictor of residual astigmatic error, and the demonstration that toric IOL accuracy is preserved in eyes targeted for intentional myopia, represent clinically relevant contributions that may inform surgical planning, calculation performance, and patient counseling in this challenging population. Further prospective studies with standardized vector analysis methodology are warranted to confirm and extend these findings.

## Figures and Tables

**Table 1:** Demographic and baseline clinical characteristics.

Variable	Value
Patients (*n*)	118
Eyes (*n*)	161
Patient characteristics (patient-based)	
Age (years, mean ± SD)	64.1 ± 15.1
Age (years, median, range)	69.1 (23–95)
Female sex (*n*%)	69 (58.5%)
Eye Distribution	
Unilateral cases (*n*%)	75 (63.6%)
Bilateral cases (*n*%)	43 (36.4%)
Surgical indication (eye-based)	
Cataract surgery (*n*%)	133 (82.6%)
Refractive lens exchange (*n*%)	28 (17.4%)
Target Refraction (eye-based)	
Emmetropia target (*n*%)	113 (70.2%)
Minus target (*n*%)	48 (29.8%)
Preoperative corneal astigmatism orientation (eye-based)	
With-the-rule (*n*%)	84 (52.2%)
Against-the-rule (*n*%)	65 (40.4%)
Oblique (*n*%)	12 (7.5%)
Preoperative corneal astigmatism magnitude (eye-based)	
Astigmatism (mean D ± SD)	2.66 ± 1.23
Astigmatism (median [IQR])	2.37 [1.93–3.08]
Astigmatism ≥ 3.00 D (*n*%)	42 (26.1%)
Astigmatism ≥ 4.00 D (*n*%)	18 (11.2%)

Data is presented as specified either by patient (patient-based) or per analyzed eye (eye-based). *n*—number of, SD—Standard Deviation, D—Diopter, IQR—Interquartile range.

**Table 2:** Preoperative ocular and biometric characteristics (eye-based).

Variable	Mean ± SD	Median [IQR]	*n*
K1 Flat (mean D ± SD)	42.67 ± 1.83	42.59 [41.26–44.04]	161
K2 Steep (mean D ± SD)	45.33 ± 1.97	45.31 [44.02–46.64]	161
Corneal Astigmatism D	2.66 ± 1.23	2.37 [1.93–3.08]	161
Axial Length (mm)	24.73 ± 2.40	24.19 [23.02–25.73]	161
Anterior Chamber Depth (mm)	3.47 ± 0.58	3.45 [3.06–3.81]	161
Central Corneal Thickness (µm)	545.35 ± 36.09	541 [524–563]	161
Lens thickness (mm)	4.53 ± 0.43	4.50 [4.22–4.82]	159
White-to-White (mm)	11.68 ± 0.46	11.69 [11.44–11.93]	158
Axial Length > 26 mm (*n*%)	38 (23.6%)	-	161

Values are presented as mean ± standard deviation (SD) and median [interquartile range, IQR], unless otherwise specified. Corneal astigmatism was calculated as the difference between steep and flat keratometry (ANTERION^®^). Axial length > 26 mm was considered indicative of high myopia. The number of eyes (*n*) may vary between variables due to missing measurements, primarily in eyes with dense cataract, where optical biometry was not feasible, and alternative measurement methods were used. D—Diopter, SD—Standard Deviation, mm—millimeter, µm—microns, IQR—Interquartile Range, K1—Flat Keratometry, K2—Steep Keratometry, *n*—number of.

**Table 3:** Surgical and intraoperative characteristics (eye-based).

Variable	*n* (*n*%)
Intraocular Lens (IOL) Category	
Monofocal Toric IOLs	127 (78.9%)
Presbyopia-correcting Toric IOLs (trifocal and extended depth of field)	34 (21.1%)
Intraocular Lens Model	
Alcon AcrySof IQ Toric	88 (54.7%)
Alcon Clareon Toric	19 (11.8%)
Zeiss AT TORBI	20 (12.4%)
Zeiss AT LISA TriToric	20 (12.4%)
Alcon PanOptix Toric	4 (2.5%)
Alcon Vivity Toric	7 (4.3%)
SIFI MiniWell Toric	3 (1.9%)
Toric IOL Cylinder power (Diopters, measured at IOL plane)	
≤2.00 D	10 (6.2%)
>2.00 D up to 3.00 D	97 (60.2%)
>3.00 D	54 (33.5%)
Use of Capsular Tension Ring	
Capsular Tension Ring Used	28 (17.4%)
Location of Main Paracentesis	
Standard—170–180° (0–10°)	132 (82.0%)
On the axis of astigmatism meridian	29 (18.0%)
Additional Procedures	
Intravitreal anti-VEGF injection	2 (1.2%)
Pupilloplasty	1 (0.6%)
Corneal suture at main paracentesis	1 (0.6%)
IOL cylinder power calculation method	
Kane toric calculator	76 (47.2%)
Barrett toric calculator	45 (28%)
Zeiss Calculator	40 (24.8%)

Values are presented as the number of eyes (*n*) and percentage (%). Percentages are calculated relative to the total number of eyes. Toric IOL cylinder power refers to the cylinder at the IOL plane as specified by the implanted lens. The cylindrical power calculation method refers to the formula or calculator used for cylindrical IOL power selection (formulas for spherical power are detailed in Materials and Methods); toric power and alignment were determined using the corresponding manufacturer-specific or dedicated toric calculators. IOL—Intraocular lens, D—Diopter, VEGF—vascular endothelial growth factor.

**Table 4:** Summary of visual and refractive outcomes.

Variable	Preoperative	Postoperative (1 Month)	*n*
Visual Acuity (logMAR)			
UDVA	1.29 ± 0.67	0.19 ± 0.24	161
CDVA	0.55 ± 0.51	0.09 ± 0.15	161
Visual Acuity Sensitivity Analysis (logMAR, excluding eyes with known preoperative reduced visual potential)			
UDVA	1.12 ± 0.59	0.11 ± 0.15	126
CDVA	0.45 ± 0.42	0.03 ± 0.05	126
Refraction			
Sphere	−3.15 ± 6.24 (*n* = 130)	−0.41 ± 1.32 (*n* = 161)	
Cylinder (minus notation)	−2.80 ± 1.29 (*n* = 130)	−0.52 ± 0.49 (*n* = 161)	
Spherical Equivalent (SE)	−4.55 ± 6.33 (*n* = 130)	−0.68 ± 1.29 (*n* = 161)	
Safety and Efficacy Indexes			
Safety Index	-	2.58 ± 2.19	95
Efficacy Index	-	0.84 ± 0.24	161
Efficacy Index for plano-target eyes only	-	0.87 ± 0.21	113
Refractive accuracy (relative to intended target)			
Outcome	*n* (*n*%)		
Spherical Equivalent within ± 0.50 D	112 (69.6%)		
Spherical Equivalent within ± 1.00 D	158 (98.1%)		
Prediction Error			
Outcome	Mean ± SD		
Mean Spherical Equivalent Error (D)	−0.19 ± 0.42		
Median Spherical Equivalent Error (D)	−0.18		
Residual Astigmatism			
Outcome	*n* (*n*%)		
Residual cylinder ≤ 0.50 D	100 (62.1%)		
Residual cylinder ≤ 1.00 D	143 (88.8%)		

Values are presented as mean ± standard deviation (SD) unless otherwise specified. Percentages are calculated relative to the total number of eyes. Preoperative refractive data were available for a subset of eyes (*n* = 130) due to the presence of dense cataracts precluding reliable refraction in some cases. Postoperative refractive and visual acuity data were available for all eyes. Low-vision measurements (counting fingers, hand motion, and light perception) were converted to logMAR equivalents for analysis but excluded from the Safety Index calculation. Values for eyes without visually limiting comorbidities are presented as a sensitivity analysis (*n* = 126). Cylinder values are presented in minus cylinder notation. Residual astigmatism is reported as the absolute magnitude of the postoperative refractive cylinder. Refractive accuracy and prediction error are calculated relative to the intended postoperative refractive target for each eye. UDVA—uncorrected distance visual acuity, CDVA—corrected distance visual acuity, SE—spherical equivalent, logMAR—logarithm of the minimum angle of resolution, D—diopters, *n*—number of.

**Table 5:** Corneal-plane vector analysis of astigmatic outcomes (Alpins method [23]).

Variable	Mean ± SD	Median [IQR]	*n*
TIA Magnitude (D)	2.76 ± 1.39	2.47 [1.98–3.18]	161
SIA Magnitude (D)	2.88 ± 1.43	2.59 [2.05–3.28]	161
Difference Vector (DV) (D)	0.53 ± 0.44	0.42 [0.22–0.74]	161
Correction Index (CI)	1.04 ± 0.20	1.01 [0.92–1.13]	161
Index of Success (IOS)	0.22 ± 0.18	0.18 [0.07–0.29]	161
Angle of Error (°)	−0.15 ± 5.37	0.00 [−2.32–1.99]	161
Performance Distribution			
Category	Eyes (*n*)	Eyes (*n*%)	
<0.9 (Undercorrection)	22	13.7%	
0.9–1.1 (On Target)	81	50.3%	
>1.1 (Overcorrection)	58	36.0%	
Angle of Error (AE)			
Category	Eyes (*n*)	Eyes (*n*%)	
Within ± 5°	114	70.8%	
Within ± 10°	148	91.9%	
Within ± 15°	157	97.5%	

Vector analysis was performed at the corneal plane according to the Alpins Method [23]. A separate sensitivity analysis presented in Table A2 excludes two patients with intraoperative events that could influence astigmatism readings (posterior capsule rupture, corneal suture). This analysis quantifies exact measurement differences and demonstrates the robustness of vectorial analysis results. Preoperative corneal astigmatism data was obtained from tomo-topographic keratometry (corneal plane) with ANTERION^®^ biometer, and target and postoperative refractive astigmatism were converted from spectacle plane (vertex distance 12 mm) to corneal plane prior to analysis. Values are presented as Mean ± Standard Deviation (SD) and Median [Interquartile Range], unless otherwise specified. SD—Standard Deviation, D—Diopter, IQR—Interquartile Range, TIA—Target Induced Astigmatism, SIA—Surgically Induced Astigmatism, DV—Difference Vector, CI—Correction Index (SIA/TIA), IOS—Index of Success (DV/TIA).

**Table 6:** Spectacle Independence and Strategy-Based Outcomes (Patient-based Analysis).

Section	Category/Group	Patients (*n*)	Patients (*n*%)	*p*-Value
Overall spectacle status	Complete spectacle independence	18	15.3	-
	Spectacle dependent *	100	84.7	-
Detailed spectacle dependence profile	None (independent)	18	15.3	-
	Reading only	55	46.6	-
	Distance only	30	25.4	-
	Distance + near	13	11.0	-
	Mixed bilateral status **	2	1.7	-
Achievement of intended outcome (strategy-based)	Minus-Target monofocal (*n* = 34): success (distance only)	29	85.3	0.167 ***
	Monofocal emmetropia-target (*n* = 62): success (reading only)	45	72.6	
	Presbyopia-correcting IOL (*n* = 22): success (no glasses)	14	63.6	

* Spectacle-dependent includes patients requiring distance and/or near correction. ** Patients with discordant spectacle dependence between eyes were classified as spectacle dependent. *** Chi-Square X^2^ Test. Success was defined as achievement of the intended postoperative functional outcome for each refractive strategy: distance-only spectacle use for minus-target eyes, reading-only spectacle use for emmetropia-target monofocal intraocular lens (IOL) eyes, and complete spectacle independence for presbyopia-correcting IOL eyes. Percentages in the overall sections are calculated relative to the total cohort (*n* = 118), whereas strategy-specific success rates are calculated within each subgroup. IOL—Intraocular Lens, RLE—Refractive Lens Exchange.

**Table 7:** Multivariate linear regression analysis of predictors of residual astigmatic error (Difference Vector Magnitude).

Variable	β	Standard Error	95% Confidence Interval	*p*-Value
IOL Cylinder power (D)	0.051	0.023	0.005–0.096	0.031
CTR use (vs. no CTR)	0.145	0.095	−0.043–0.333	0.130
ATR Astigmatism (vs. WTR)	0.071	0.075	−0.077–0.219	0.343
Oblique Astigmatism (vs. WTR)	0.299	0.138	0.027–0.570	0.032

Model summary: *n* = 161 eyes; R^2^ = 0.089; Adjusted R^2^ = 0.066; F (4156) = 3.81; *p* = 0.005. Outcome Variable: Difference Vector (DV), magnitude (D), representing residual astigmatic error at the corneal plane. Candidate predictors were screened by univariate linear regression; variables with *p* < 0.20 were entered into the multivariate model. Reference categories: WTR (with-the-rule) astigmatism, no CTR use. Β—Unstandardized regression coefficient; D—Diopters; IOL—Intraocular lens; CTR—Capsular tension ring; ATR—Against-the-rule astigmatism.

**Table 8:** Refractive stability analysis.

Follow-Up Characteristics	Value
Total eyes with extended follow-up	*n* = 75
Mean follow-up (months)	8
Median follow-up (months)	7
Maximal follow-up (months)	25
**Refractive stability Outcomes**	**Value**
Mean SE at 1 month (D ± SD)	−0.71 ± 1.24
Mean SE at extended follow-up (D ± SD)	−0.68 ± 1.22
Mean SE change (extended—1 month) ± SD	0.02 ± 0.31
Eyes with SE change ≤ 0.50 D	68
% with SE change ≤ ±0.50 D	90.7%
Duration Category	Eyes *n* (*n*%)
2–6 months	34 (44%)
7–12 months	30 (40%)
13–18 months	6 (8%)
>18 Months	5 (6.7%)

SE—Spherical Equivalent, D—Diopter, SD—Standard Deviation.

**Table 9:** Comparison of astigmatic correction outcomes between monofocal emmetropia-targeted and monofocal minus-targeted eyes.

Variable	Emmetropia Target (*n* = 78 Eyes)	Minus-Target (*n* = 48 Eyes)	*p*-Value
**A. Astigmatic Vector Metrics (Alpins method** [23]**, corneal plane)**			
DV Magnitude Median D. (IQR)	0.447 (0.202–0.747)	0.348 (0.134–0.673)	0.310
Correction Index Median (IQR)	1.019 (0.944–1.195)	0.975 (0.923–1.066)	0.049
Angle of Error Mean ± SD	0.02 ± 4.82	0.19 ± 5.18	0.858 ^1^
**B. Residual Postoperative Astigmatism**			
Residual Cylinder Median D. (IQR)	0.500 (0.250–0.750)	0.375 (0.00–0.750)	0.370
≤0.50 D Residual Cylinder *n* (*n*%)	52 (66.7%)	34 (70.8%)	0.771 ^2^
≤1.00 D Residual Cylinder *n* (*n*%)	71 (91.0%)	44 (91.7%)	1.000 ^3^
**C. Spherical Equivalent Prediction Error**			
SE-PE Median D. (IQR)	−0.185 (−0.499 to +0.081)	−0.165 (−0.461 to +0.125)	0.746
**D. Visual Outcome**			
Postoperative CDVA Median logMAR (IQR)	0.04 (0.00–0.10)	0.04 (0.00–0.16)	0.077

Continuous variables are presented as median (interquartile range, IQR) unless otherwise specified; Angle of Error (AE) is reported as mean ± standard deviation (SD). Between-group comparisons were performed using the Mann–Whitney U test for continuous variables, with the exception of: ^1^ independent samples *t*-test for AE. For categorial values comparison the following were used: ^2^ Chi-square test, ^3^ Fisher’s exact test. Emmetropia target was defined as monofocal IOL implantation with intended postoperative spherical equivalent (SE) ≥ −0.50 D. Minus target was defined as monofocal IOL implantation with intended residual myopia (−1.00 D to −2.50 D) to preserve uncorrected near and/or intermediate vision. DV—Difference Vector; D—Diopters; IQR—Interquartile Range; SD—Standard Deviation; AE—Angle of Error; SE-PE—Spherical Equivalent Prediction Error; CDVA—Corrected Distance Visual Acuity; logMAR—Logarithm of the Minimum Angle of Resolution; IOL—Intraocular Lens.

**Table 10:** Comparison of visual, refractive, and vector outcomes between cataract surgery and refractive lens exchange (RLE).

Variable	Cataract Patients (*n* = 133 Eyes)	RLE Patients (*n* = 28 Eyes)	*p*-Value
**Visual Acuity**			
Postoperative UDVA mean logMAR ± SD	0.21 ± 0.25	0.12 ± 0.18	0.043
Postoperative UDVA median logMAR [IQR]	0.10 [0.00–0.30]	0.00 [0.00–0.22]	
Postoperative CDVA mean logMAR ± SD	0.09 ± 0.15	0.05 ± 0.11	0.031
Postoperative CDVA median logMAR [IQR]	0.05 [0.00–0.10]	0.00 [0.00–0.05]	
**Spherical Refractive Accuracy**			
SE Prediction Error mean D ± SD	−0.18 ± 0.41	−0.20 ± 0.45	0.836
SE Prediction Error median D [IQR]	−0.18 [−0.46 to +0.06]	−0.20 [−0.50 to +0.10]	
**Astigmatic Vector Analysis (Alpins method, corneal plane)**			
Difference Vector mean D ± SD	0.52 ± 0.42	0.57 ± 0.53	0.646
Difference Vector median D [IQR]	0.40 [0.20–0.77]	0.47 [0.25–0.69]	
Correction Index mean ± SD	1.04 ± 0.20	1.01 ± 0.18	0.254
Correction Index median [IQR]	1.02 [0.92–1.15]	0.97 [0.91–1.11]	
Index of Success mean ± SD	0.22 ± 0.18	0.21 ± 0.17	0.805
Index of Success median [IQR]	0.18 [0.07–0.30]	0.18 [0.08–0.29]	
Angle of Error mean ± SD	+0.17 ± 5.36	−1.69 ± 5.22	0.095 ^1^
**Residual Astigmatism (corneal plane)**			
Residual Cylinder mean D ± SD	0.50 ± 0.47	0.58 ± 0.56	0.504
Residual Cylinder median D [IQR]	0.47 [0.23–0.76]	0.50 [0.25–0.75]	
Residual Cylinder ≤ 0.50 D (*n*%)	81 (60.9%)	19 (67.9%)	0.529 ^2^
Residual Cylinder ≤ 1.00 D (*n*%)	117 (88.0%)	26 (92.9%)	0.741 ^2^

Continuous variables compared using the Mann–Whitney U test unless otherwise specified. ^1^ Angle of error compared using an independent samples *t*-test. ^2^ Fisher’s exact test. All astigmatic outcome measures were reported at the corneal plane (vertex distance 0 mm). Values presented as mean ± SD (median [IQR]) where applicable. RLE, Refractive Lens Exchange; UDVA, Uncorrected Distance Visual Acuity; CDVA, Corrected Distance Visual Acuity; SD, Standard Deviation; IQR, Interquartile Range; logMAR, Logarithm of the Minimum Angle of Resolution; SE, Spherical Equivalent; D—Diopters.

**Table 11:** Intraoperative Events and Postoperative Complications (Eye-based, *n* = 161).

Event	Eyes *n*	Eyes *n*%	Notes
**Intraoperative**			
Posterior Capsule Rupture with anterior vitrectomy	1	0.6%	No impact on planned IOL implantation
Corneal Suture at Main Incision	1	0.6%	Prophylactic measure, for added safety
Intravitreal anti-VEGF Injection	2	1.2%	Planned combined procedure for pre-existing macular pathology
Pupilloplasty	1	0.6%	Traumatic cataract case
Synechiolysis	1	0.6%	Uveitis Sequelae
**Postoperative period (up to 1 month)**			
Transient Corneal Edema	10	6.2%	Transient, resolved by 1 month in all cases
IOP Elevation	4	2.5%	Managed topically
Dry Eye/Ocular Surface Symptoms	3	1.9%	
Conjunctivitis	1	0.6%	Resolved without sequelae
Toric IOL Rotation ≥ 15°	2	1.2%	No repositioning required
Posterior Capsule Opacification	7	4.3%	4 cases treated with Nd:YAG capsulotomy at 1-month postop
Intraoperative Complications	1	0.6%	
Any Postoperative Complications	27	16.8%	
**Postoperative Summary**			
Eyes with Complications at 1 month	17	10.6%	Excluding resolved corneal edema
No Complications at 1 month	142	88.2%	

IOP—Intraocular pressure, VEGF—Vascular Endothelial Growth Factor, Nd:YAG—Neodymium-Doped Yttrium Aluminum Garnet (Laser).

## Data Availability

This paper presents all the resultant statistical data from the study. Datasets produced and analyzed by the study are available upon request.

## Abbreviations

The following abbreviations are used in this manuscript:

IOL	Intraocular Lens
RLE	Refractive Lens Exchange
D	Diopter
UDVA	Uncorrected Distance Visual Acuity
CDVA	Corrected Distance Visual Acuity
logMAR	Logarithm of the minimum angle of resolution
WTR	With-the-rule (astigmatism)
ATR	Against-the-rule (astigmatism)
FSAK	Femtosecond laser-assisted astigmatic keratotomy
CTR	Capsular Tension Ring
SD	Standard Deviation
*n*	Number of
Pt(s)	Patient (s)
LASIK	Laser-Assisted In Situ Keratomileusis
LASEK	Laser-Assisted Epithelial Keratomileusis
RK	Radial Keratotomy
VD	Vertex distance
mm	Millimeters
µm	Microns
OCT	Optical Coherence Tomography
K1	Flat keratometry (K flat)
K2	Steep keratometry (K steep)
AL	Axial length
ACD	Anterior Chamber Depth
AQD	Aqueous Depth
CCT	Central Corneal Thickness
LT	Lens Thickness
WTW	White-to-White (Diameter)
ESCRS	European Society of Cataract and Refractive Surgeons
Anti-VEGF	Anti-Vascular Endothelial Growth Factor
ASCRS	American Society of Cataract and Refractive Surgery
IQR	Interquartile Range
SE	Spherical Equivalent
SI	Safety Index
EI	Efficacy Index
DEQ	Defocus Equivalent
AE	Angle of Error
TIA	Target Induced Astigmatism
SIA	Surgically Induced Astigmatism
DV	Difference Vector
CI	Correction Index
IOS	Index of Success
RA-PE	Refractive Astigmatism Prediction Error
Nd:YAG	Neodymium-Doped Yttrium Aluminum Garnet (laser)
IOLP	Intraocular Lens Power (calculated via Biometry)
LED	Light-emitting diode

## Appendix A

**Table A1:** Univariate linear regression screening of candidate predictors of residual astigmatic error (difference vector magnitude, corneal plane).

Variable	Univariate *p*-Value	Entered Multivariate Model (Variables Achieving *p* < 0.20 Were Entered into the Multivariate Model)
IOL cylinder power (D)	0.003	Yes
Astigmatism orientation (WTR/ATR/oblique)	0.041 ^1^	Yes
Capsular tension ring use	0.162	Yes
Preoperative corneal astigmatism magnitude (D)	>0.32	No
Axial length (mm)	>0.32	No
Age (years)	>0.32	No
Surgery type (cataract vs. RLE)	>0.32	No
Intended refractive target (emmetropia vs. myopia)	>0.32	No

Variables were screened by simple linear regression against the difference vector magnitude (corneal plane) as the outcome. The threshold for entry into the multivariate model was *p* < 0.20. ^1^ Astigmatism orientation was evaluated as a categorical variable using one-way ANOVA (WTR as reference category). Abbreviations: IOL, intraocular lens; D, diopters; WTR, with-the-rule; ATR, against-the-rule; RLE, refractive lens exchange.

**Table A2:** Sensitivity analysis of vector and refractive astigmatic outcomes after exclusion of eyes with astigmatism-influencing intraoperative events (posterior capsule rupture and corneal suture) (*n* = 159).

Variable	Sensitivity Analysis (*n* = 159)	Primary Analysis (*n* = 161)	Difference from Excluding Two Patients
**Vector Analysis (Alpins method, corneal plane)**			
TIA mean D ± SD (median)	2.70 ± 1.08 (2.47)	2.76 ± 1.39 (2.47)	+0.06 ± 0.31
SIA, mean D ± SD (median)	2.82 ± 1.17 (2.59)	2.88 ± 1.43 (2.59)	+0.06 ± 0.26
Difference Vector, mean D ± SD (median)	0.53 ± 0.44 (0.41)	0.53 ± 0.44 (0.42)	Only median differs by 0.01
Correction Index, mean ± SD (median)	1.04 ± 0.19 (1.01)	1.04 ± 0.20 (1.01)	No difference
Index of Success, mean ± SD (median)	0.22 ± 0.18 (0.18)	0.22 ± 0.18 (0.18)	No difference
Angle of Error mean ° ± SD (median)	−0.05 ± 5.11 (0.00)	−0.15 ± 5.37 (0.00)	+0.10 ± 0.26
**Residual Astigmatism (spectacle plane)**			
Residual Cylinder mean D ± SD (median)	0.51 ± 0.47 (0.48)	0.52 ± 0.49 (0.47)	−0.01 ± 0.02 median differs by 0.01
Residual Cylinder ≤ 0.50 D, *n* (%)	105 (66.0%)	100 (62.1%)	Gain of 3.9% for excluding the 2 patients
Residual Cylinder ≤ 1.00 D, *n* (%)	147 (92.5%)	143 (88.8%)	Gain of 3.7% for excluding the 2 patients

Sensitivity analysis excludes one eye with posterior capsule rupture managed with anterior vitrectomy and one eye with a prophylactic corneal suture at the main incision (*n* = 159). In the posterior capsule rupture case, the planned toric IOL was successfully implanted in the capsular bag with no documented impact on IOL positioning. Results are presented alongside the primary analysis (*n* = 161) to demonstrate the robustness of the findings. TIA, Target Induced Astigmatism; SIA, Surgically Induced Astigmatism; D, Diopters; SD, Standard Deviation; IOL, Intraocular Lens.
